# Artemisinin attenuates 3-nitropropionic acid-induced neurodegeneration via HMGB1/TLR4/NF-κB modulation in a rat model of huntington’s disease

**DOI:** 10.1007/s12272-026-01604-1

**Published:** 2026-03-22

**Authors:** Aya M. Mustafa, Mustafa Mudhafar, Ali M. Elgindy, Manar M. Esmail, Ahmed M. Atwa, Aya M. Shaheen, Abdullah F. Radwan, Nourhan Elfar, Ruaa Yasir Altayeb Mohamed, Noha M. Gamil

**Affiliations:** 1 Department of Pharmacology and Toxicology, Faculty of Pharmacy, Egyptian Russian University, Badr City, Cairo, 11829 Egypt; 2 Centre for Research on Environment and Renewable Energy, University of Karbala, 56001 Karbala, Iraq; 3 Department of Anesthesia Techniques and Intensive Care, Al-Taff University College, 56001 Kerbala, Iraq; 4 College of Pharmacy, Al-Ayen Iraqi University, AUIQ, An Nasiriyah, Iraq; 5 Department of Biochemistry, Faculty of Pharmacy, Egyptian Russian University, Badr City, Cairo, 11829 Egypt; 6 College of Pharmacy, University of Kut, 52001 Wasit, Iraq; 7 Department of Biochemistry, School of Health and Social Work, University of Hertfordshire Hosted By Global Academic Foundation, New Administrative Capital, 11578 Cairo Egypt; 8 Department of Pharmacology and Toxicology, College of Pharmaceutical Sciences and Drug Manufacturing, Misr University for Science and Technology, Giza, Egypt

**Keywords:** Huntington’s disease, 3-nitropropionic acid, Artemisinin, Neuroinflammation, HMGB1/TLR4/NF-κB, Necroptosis

## Abstract

Huntington’s disease (HD) is a progressive neurodegenerative disorder characterized by motor, cognitive, and behavioral impairments associated with striatal neuronal loss, for which effective symptom-attenuating therapies remain lacking. Artemisinin (ART), a natural sesquiterpene lactone with established antioxidant and anti-inflammatory actions, has recently gained attention as a potential neuroprotective agent. This study evaluated the therapeutic relevance of ART in a rat model of HD induced by 3-nitropropionic acid (3-NP). 3-NP administration caused severe behavioral deficits, including an 81.8% reduction in rearing and a 74.9% reduction in ambulation (p < 0.0001), a 63.7% decrease in novel object exploration, and a 53.5% decline in Morris water maze target quadrant time versus controls. Biochemically, 3-NP elevated HMGB1 (4.8-fold), TLR4 (6.8-fold), RIPK1 (6.4-fold), RIPK3 (5.2-fold), MLKL (5.5-fold), p38-MAPK (4.2-fold), NF-κB (2.1-fold), and TNF-α (4.5-fold), while reducing GSH (57.6%), Nrf2 (77.7%), Sig1R (86.2%), D2R (64%), XIAP (77.8%), BDNF (57.6%) and SDH (61.44%) (all p < 0.0001). Treatment with ART (100 mg/kg) markedly restored behavioral performance, increasing rearing and ambulation by 3.2- and 2.6-fold, novel object exploration by 2.4-fold, and target quadrant time by 1.7-fold compared to the 3-NP group. At the molecular level, ART reduced HMGB1 (69.2%), TLR4 (60.4%), RIPK1 (66.3%), RIPK3 (66.4%), MLKL (58%), and TNF-α (62.5%), while significantly restoring GSH (2.1-fold), Nrf2 (3.7-fold), Sig1R (5.2-fold), D2R (2.6-fold), XIAP (3.7-fold), BDNF (2.3-fold) and SDH (1.94-fold) relative to 3-NP-treated rats. Collectively, these results demonstrate that ART confers robust neuroprotection against 3-NP-induced HD-like pathology by attenuating oxidative stress, suppressing HMGB1/TLR4/NF-κB signaling, inhibiting necroptosis, and upregulating neuroprotective markers. These findings highlight ART not only as a neuroprotective agent but also as a promising symptom-attenuating therapeutic candidate for Huntington’s disease and other neurodegenerative disorders driven by oxidative and inflammatory stress.

**Supplementary Information:**

The online version contains supplementary material available at 10.1007/s12272-026-01604-1.

## Introduction

Huntington’s disease (HD) is a neurodegenerative disorder marked by progressive loss of striatal neurons, leading to impaired motor function and cognitive decline (Cho 2012). Clinically, HD presents with uncontrollable movements, such as chorea, along with cognitive deficits and behavioral disturbances (Kumar et al. 2010). One of the key pathological features of HD is mitochondrial dysfunction, particularly involving impaired energy metabolism (Gao et al. 2015). To investigate the underlying mechanisms of HD-related neurodegeneration, 3-nitropropionic acid (3-NP), a mitochondrial toxin, is commonly used as an experimental model. 3-NP induces striatal damage and motor impairments similar to those observed in HD, making it a valuable tool for studying disease pathophysiology and potential therapeutic interventions (Wu et al. 2010; Ibrahim and Abdel Rasheed 2022).

Neuroinflammation is another key feature of HD progression. The release of damage-associated molecular patterns (DAMPs), particularly high-mobility group box 1 (HMGB1), activates Toll-like receptor 4 (TLR4), triggering downstream nuclear factor-κB (NF-κB) and mitogen-activated protein kinase (MAPK) pathways (Mustafa et al. 2024). This results in sustained oxidative stress, production of proinflammatory cytokines, and neuronal death. Furthermore, receptor-interacting kinases receptor-interacting protein kinase (RIPK1) and RIPK3 act as central mediators of necroptosis, a regulated form of necrotic cell death that exacerbates striatal injury and fuels inflammatory feedback loops (Wei et al. 2023). The interplay between mitochondrial dysfunction, oxidative stress, and necroptotic signaling provides a strong rationale for targeting these pathways in HD.

Artemisinin (ART), a sesquiterpene lactone derived from Artemisia annua, is well established as a frontline antimalarial agent. However, accumulating evidence highlights its broader pharmacological actions, including potent antioxidant, anti-inflammatory, and antiapoptotic properties (Zhao et al. 2019; Lin et al. 2021). Preclinical studies have demonstrated that ART can modulate TLR4/NF-κB signaling and suppress proinflammatory cytokine release (Zhao et al. 2022). Apart from its anti-inflammatory properties, ART has been demonstrated to boost the synthesis of antiapoptotic proteins, an especially cellular inhibitor of apoptosis (c-IAP-1, c-IAP-2, and xIAP) (Xu et al. 2017a, b), which are known to control RIPK1/RIPK3 levels by promoting their ubiquitination and degradation, so inhibit apoptosis and necroptosis (Lawlor et al. 2015; Ali et al. 2021). Moreover, in models of Alzheimer’s and Parkinson’s disease, ART treatment has been shown to improve behavioral outcomes and restore neuroprotective markers, supporting its therapeutic relevance beyond infectious disease (Zhao et al. 2019).

Given these findings, we hypothesized that ART could mitigate striatal degeneration and behavioral deficits in HD by targeting oxidative stress, neuroinflammation, and necroptosis pathways. Therefore, the present study aimed to evaluate the neuroprotective potential of ART in a 3-NP-induced rat model of HD, with a particular focus on its effects on HMGB1/TLR4/NF-κB signaling and RIPK1/RIPK3-mediated necroptosis.

## Materials and methods

### Animals

Sixty male albino rats, ranging in weight from 180 to 200 grammes, were used in this research. Located in Cairo, Egypt, El-Nile Company for Pharmaceutical and Chemical Industries supplied the animals. They were given a week to become used to the lab environment before the experiment. The rats were housed in controlled conditions with a 12-h light–dark cycle, humidity maintained at 60 ± 10%, and a constant temperature of 23 ± 2 ℃. A basic chow meal and unlimited water were provided. All experimental protocols involving laboratory animals were reviewed and approved by the Research Ethics Committee of the Faculty of Pharmacy at Egyptian Russian University (Code ERUFP-PO-25-004).

### Experimental design

Rats were randomly divided into five groups (n = 12 per group). Group I (normal control) received oral saline. Group II was administered ART (100 mg/kg/day, p.o.; Sigma-Aldrich, MO, USA) (Guang Xu et al. 2017a, b), Groups III-V received 3-nitropropionic acid (3-NP; 10 mg/kg/day, i.p.; Sigma-Aldrich, MO, USA) (Mustafa et al. 2021). In addition, Group IV received ART (50 mg/kg/day, p.o.) and Group V received ART (100 mg/kg/day, p.o.) (Guang Xu et al. 2017a, b). Treatments were administered for 14 consecutive days. 3-NP and ART were prepared in saline and adjusted to pH 7.4 with sodium hydroxide.

**Scheme 1:**
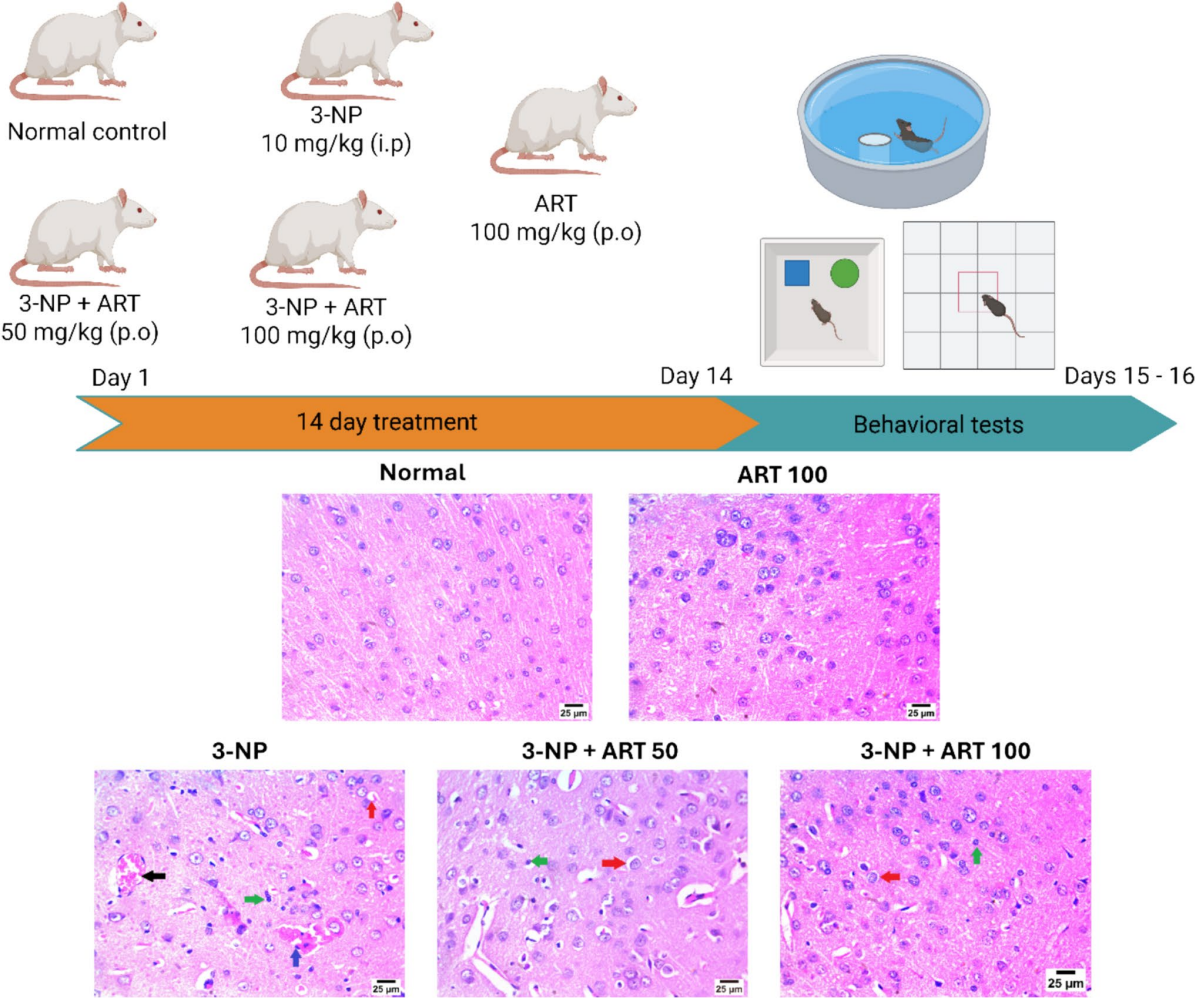
Sequence for experimental design, behavioral assessment, and histopathological alteration

The animals were tested for behavioral issues on days 15 and 16. Following the evaluations, the animals were euthanized, their striata were removed, rinsed with ice-cold saline, flash-frozen in liquid nitrogen, and stored at – 80 ℃ for future analysis. After that, three groups of brains were randomly assigned. After being immersed in RIPA buffer supplemented with protease and phosphatase inhibitors, the first fraction (n = 3/group) was used for western blot analysis. The second group, consisting of six individuals, was examined by dividing the brain into its two halves. For parameter assessment using enzyme-linked immunosorbent assay (ELISA), the striatum in the first hemisphere (n = 6) was homogenised in phosphate-buffered saline (PBS). At the same time, n = 6 of the contralateral hemisphere’s striatum were submerged in lysis buffer to evaluate parameters by RT-qPCR tests. Histological examination of the striatum and immunohistochemical research of Glial Fibrillary Acidic Protein (GFAP) were conducted on the third subgroup (n = 3/group) after preservation in 10% formalin saline (Scheme 1).

### Behavioral tests

The rats were evaluated for motor function using the open field and rotarod tests 24 h after the last injection of 3-NP and Artemisinin. The Morris water maze and the Novel Object recognition tests were also used to evaluate memory. The tests were carried out over two days, with a 2-h break between days during the light cycle. On the first day, the Morris Water Maze was administered, and on the second day, the Open Field and Novel Object Recognition tests were run (Sayed et al. 2020).

### Open field test

The open-field test was used to assess spontaneous locomotor activity. Red walls and a black polished floor formed the device’s square, with white lines dividing the box into 16 equal squares. This object measured 80 × 80 × 40 cm. The rats were scattered around the field and given five minutes to explore independently. We used the camera to monitor two behavioral variables: the number of squares each rat traversed (ambulation frequency) and the rate at which each rat stood on its hind legs (rearing frequency). After examining each animal, the floor was disinfected extensively between trials (Ramachandran and Thangarajan 2018).

### Novel object recognition test

Tests for recognition memory were administered using the Novel Object Recognition (NOR) battery. The experiment was carried out in a black open-field box measuring 50 × 25 × 50 cm. Over two days, rats were habituated by exploring the empty arena for ten minutes each day. The training phase involved placing each rat in an arena with two identical objects spaced approximately 30 cm apart. The day of the test saw the rats returned to the arena, along with the substitution of a new item for an old one. A camera above videotaped the 3-min exam and training sessions while participants explored each item (Chen et al. 2019). The discrimination index (DI) is calculated as the difference in exploration time between the novel and familiar objects divided by the total exploration time. The total time spent exploring each object was recorded (Mustafa et al. 2021).

### Morris water maze

The Morris Water Maze (MWM) was used to evaluate spatial learning and memory. The experiment was carried out in a circular pool with non-reflective inner surfaces, measuring 150 cm in diameter and 60 cm in height. The pool was filled to a depth of 35 cm with water kept at 25 ± 2 ℃. A 9-cm-diameter escape platform was placed one centimeter below the water’s surface in the middle of one of the four pool corners identified as the target area. We applied non-toxic black paint to the water to make the platform invisible. Rats underwent acquisition training for 4 consecutive days, with three trials per day (120 s per trial), beginning at different locations. If the platform could not be found during the trial, the rat was led to it and left for 30 s. The average time it took to reach the platform across trials was called acquisition latency. The platform was removed on day five to conduct a probing trial. After positioning themselves in the quadrant across from the goal, the rats were given 60 s to swim. To measure memorization, a camera mounted above the head tracked how much time was spent in each quadrant (Suganya and Sumathi 2017).

### Biochemical parameters

#### Western blot analysis of TLR4, RIPK1, RIPK3 and p38 (pT180/Y182)-MAPK

The Striatal tissues were ground in RIPA buffer, and the Bradford Protein Assay Kit (Bio-Rad, ON, Canada) was used to measure total protein content. Protein concentrations of 10 μg per sample were transferred to PVDF membranes after SDS-PAGE separation and blocked with 5% BSA. Thermo Fisher Scientific, MA, USA, provided the primary antibodies to incubate the membranes. These antibodies were specific for TLR4 TLR4 (2 μg/ml; cat#: PA5-23124), phospho-p38 MAPK (Thr180/Tyr182), (1:1000; cat#: 44-684G), RIPK1 (1 µg/mL; cat# PA5-20811), RIPK3 (0.1–0.5 µg/mL; cat# PA5-19956), and β-actin (1:1000; cat# PA5-16914). Afterward, membranes were probed with horseradish peroxidase-conjugated goat anti-rabbit immunoglobulin (Dianova, Hamburg, Germany) for 2 h at room temperature. All antibodies were validated by the supplier for specificity, and protein bands were detected at their expected molecular weights. The expression of the target protein was measured by densitometric analysis in Image Lab software on the ChemiDoc™ MP Imaging System (version 3) (Bio-Rad, CA, United States). The percentage of acrylamide used for all studied proteins was 10%. The outcomes were normalized to β-actin and presented in arbitrary units (AU).

#### ELISA assay

The following enzyme-linked immunosorbent assays (ELISA) were used to quantify BDNF, GSH, HMGB1, NF-κB p65, MDA, 8-OHdG, and SDH in phosphate-buffered saline (PBS): (cat# MBS2019439), (cat# MBS9712516), (cat# MBS703437), (cat# MBS2505513), (cat# MBS738685), (cat# MBS267513), and (cat# MBS3807968) from MyBioSource, Irvine, USA. A Cusabio ELISA kit (cat# CSB-E11987r) from Wuhan, PRC was used to test TNF-α. All tests were carried out according to the manufacturer’s instructions. Results for BDNF, NF-κB p65, TNF-α, and 8-OHdG were expressed as pg/mg of tissue protein, whereas GSH, HMGB1, MDA, and SDH levels were reported as ng/mg of tissue protein.

#### Quantitative RT-PCR

Brain tissues were homogenized for mRNA extraction. Total RNA was isolated, and cDNA was synthesized using a reverse transcription system. Quantitative RT-PCR was conducted for XIAP, Nrf2, D2R, Sig1R, and MLKL using SYBR Green Master Mix. Amplification was performed for 40 thermal cycles under optimized conditions. Primer specificity was confirmed by melt-curve analysis, which demonstrated a single sharp peak for each target, indicating the absence of non-specific amplification or primer-dimer formation.

Amplification efficiencies for all primer pairs were validated using standard curve analysis and were within the acceptable range (90–110%), allowing the use of the comparative cycle threshold (2^−ΔΔCt) method. The housekeeping gene GAPDH was selected as an internal reference, and its expression was verified to be stable across all experimental groups. Relative gene expression levels were calculated using the Ct method, normalized to GAPDH, and expressed as fold changes relative to the control group.

#### Histopathological examination

Over 72 h, with solution changes made daily, tissue samples were fixed in 10% neutral buffered formalin. Rinsing, dehydration using a graded ethanol series, xylene clearing, infiltration with synthetic wax, and embedding in Paraplast tissue embedding medium, followed by fixation. Light microscopy was used to analyze sagittal brain slices (5 µm) of the striatum that had been cut using a rotary microtome and stained with Hematoxylin and Eosin (H&E) (Sidhu et al. 2018). Using the Leica application module for histological analysis, all micrographs and data were collected with a full-HD microscope camera (Leica Microsystems GmbH, Wetzlar, Germany).

#### Immunohistochemical detection of GFAP

Paraffin-embedded sections were mounted on positively charged slides and processed using the avidin-biotin-peroxidase complex (ABC) method. Following incubation with primary monoclonal antibodies, sections were treated with reagents from the Vectastain ABC-HRP kit (Vector Labs). Antigen–antibody binding was visualized by peroxidase-mediated diaminobenzidine (DAB; Sigma) staining. Non-immune serum replaced primary and secondary antibodies in negative control slides. Immunostained sections were examined using an Olympus BX-53 microscope. Immunoreactivity was quantified by analyzing the percentage area of positive staining in 10 randomly selected fields per section using ImageJ (version 1.53t), as described by Rasband et al. (NIH, USA).

### Statistical analysis

Data are presented as mean ± standard deviation. A one-way ANOVA followed by Tukey’s multiple-comparison test was applied to most parameters. Ambulation and rearing frequency were analyzed using the Kruskal–Wallis test with Dunn’s post hoc comparisons. A two-way ANOVA was performed within groups to assess differences in exploration time between familiar and novel objects, accounting for both object type and treatment effects. Statistical analyses were conducted using GraphPad Prism version 9.

## Results

### Effect of artemisinin on behavioural and motor alterations in the 3-NP rat model

The Novel Object Recognition test assessed spatial and non-spatial memory in rats. Statistical analysis showed significant differences in both the discrimination index (F (4, 55) = 87.44, p < 0.0001) and total exploration time for novel versus familiar objects (F (4, 55) = 13.98, *p* < 0.0001).

Administration of 3-NP resulted in a significant decline in the discrimination index (− 0.18, p < 0.0001) and a (41.55%, p < 0.0001) reduction in total exploration time compared to the control group. However, cotreatment with 50 mg and 100 mg ART significantly improved the discrimination index (0.22, p < 0.0001) and (0.26, p < 0.0001), respectively, relative to the 3-NP group. Additionally, co-administration of 50 mg and 100 mg ART significantly increased the total time spent exploring both objects (1.5-fold, p = 0.0001) and (1.54-fold, p < 0.0001), respectively, compared to the diseased group.

Further analysis revealed that the 3-NP group showed a significant 63.65% reduction (*p* < 0.0001) in time spent exploring the novel object compared to controls. Treatment with ART at 50 mg and 100 mg doses significantly increased novel object exploration by 2.27-fold and 2.36-fold, respectively (*p* < 0.0001 vs. 3-NP group). No significant differences were found among groups in the time spent exploring the familiar object.

The open field test assessed locomotor and hyperactivity behaviors by measuring rearing and ambulation frequencies. Administration of 3-NP significantly reduced rearing and ambulation by 81.79% and 74.94%, respectively (*p* < 0.0001 vs. control). Treatment with 100 mg ART significantly restored rearing and ambulation frequencies by 3.17-fold (*p* = 0.0152) and 2.59-fold (*p* = 0.0073), respectively, compared to the 3-NP group.

**Fig. 1:**
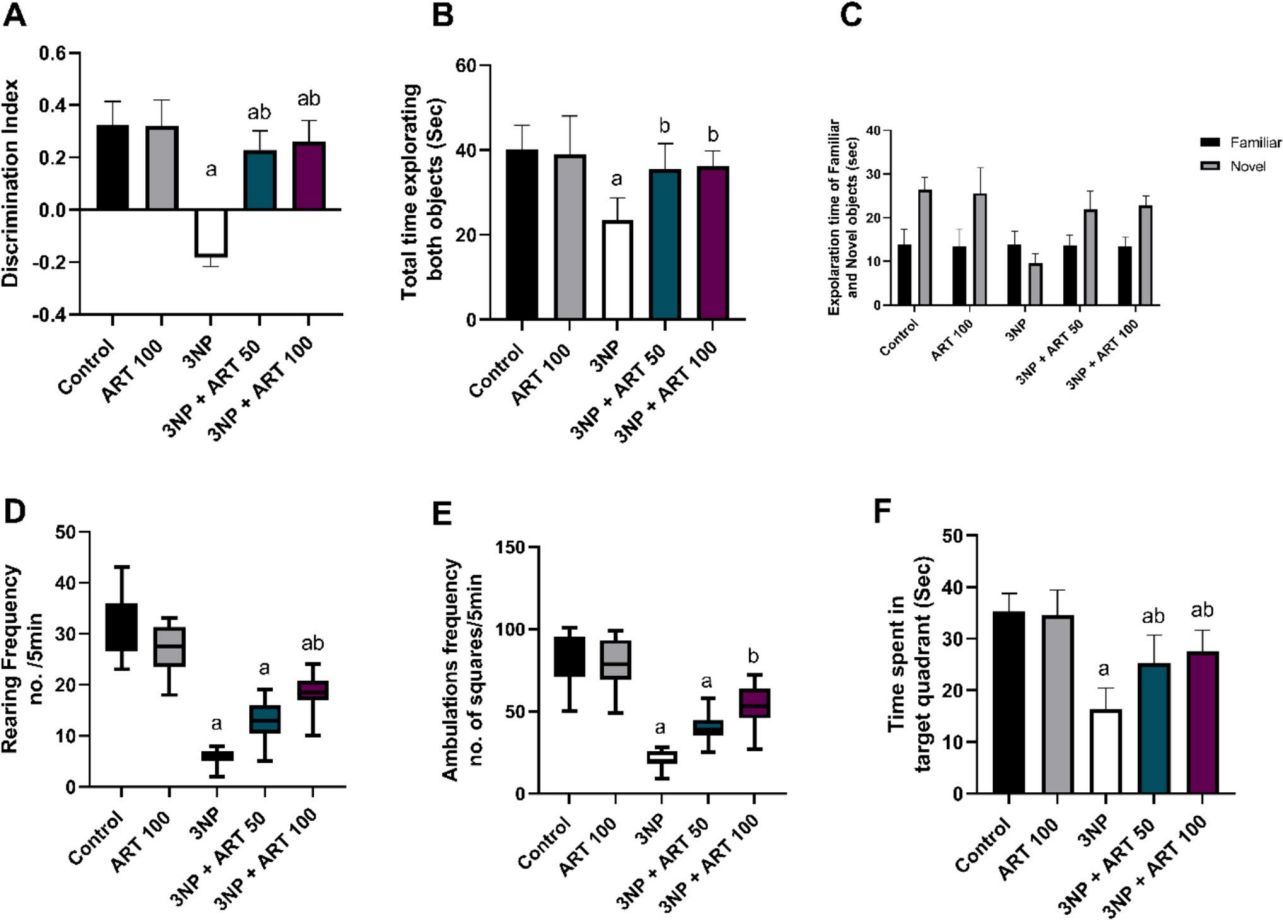
Effect of Artemisinin on 3-NP-induced alteration in **A** discrimination index, **B** Total time exploring both object and **C** the exploration time of familiar and novel objects in novel object recognition test, **E** rearing frequency and **F** ambulation frequency in Open field test, **G** time spent in target quadrant in Morris Water Maze test. Rearing & ambulation frequency are presented as boxplots with median and 25th and 75th percentile values, using the Kruskal–Wallis test followed by Dunn’s post hoc test. Parametric data are presented as mean ± SD of 12 rats per group, using one-way ANOVA followed by Tukey’s post hoc test. Differences in familiar and novel object exploration time for each group were tested using a two-way ANOVA: a vs control, b vs 3-NP, and c vs 3-NP + ART 50. 3-NP; 3-nitropropionic acid, ART; Artemisinin

The Morris Water Maze was employed to evaluate spatial memory and locomotor function. Statistical analysis revealed significant differences in time spent in the target quadrant across groups (F (4, 55) = 35.56, *p* < 0.0001). 3-NP administration significantly decreased target quadrant time by 53.47% (*p* < 0.0001) relative to controls. Treatment with ART at 50 mg and 100 mg doses significantly increased target quadrant time by 1.53-fold (*p* = 0.0001) and 1.67-fold (*p* < 0.0001), respectively, compared to the 3-NP group Fig. 1

### Effect of artemisinin on 3-NP-induced striatal histopathological alterations

**Fig. 2:**
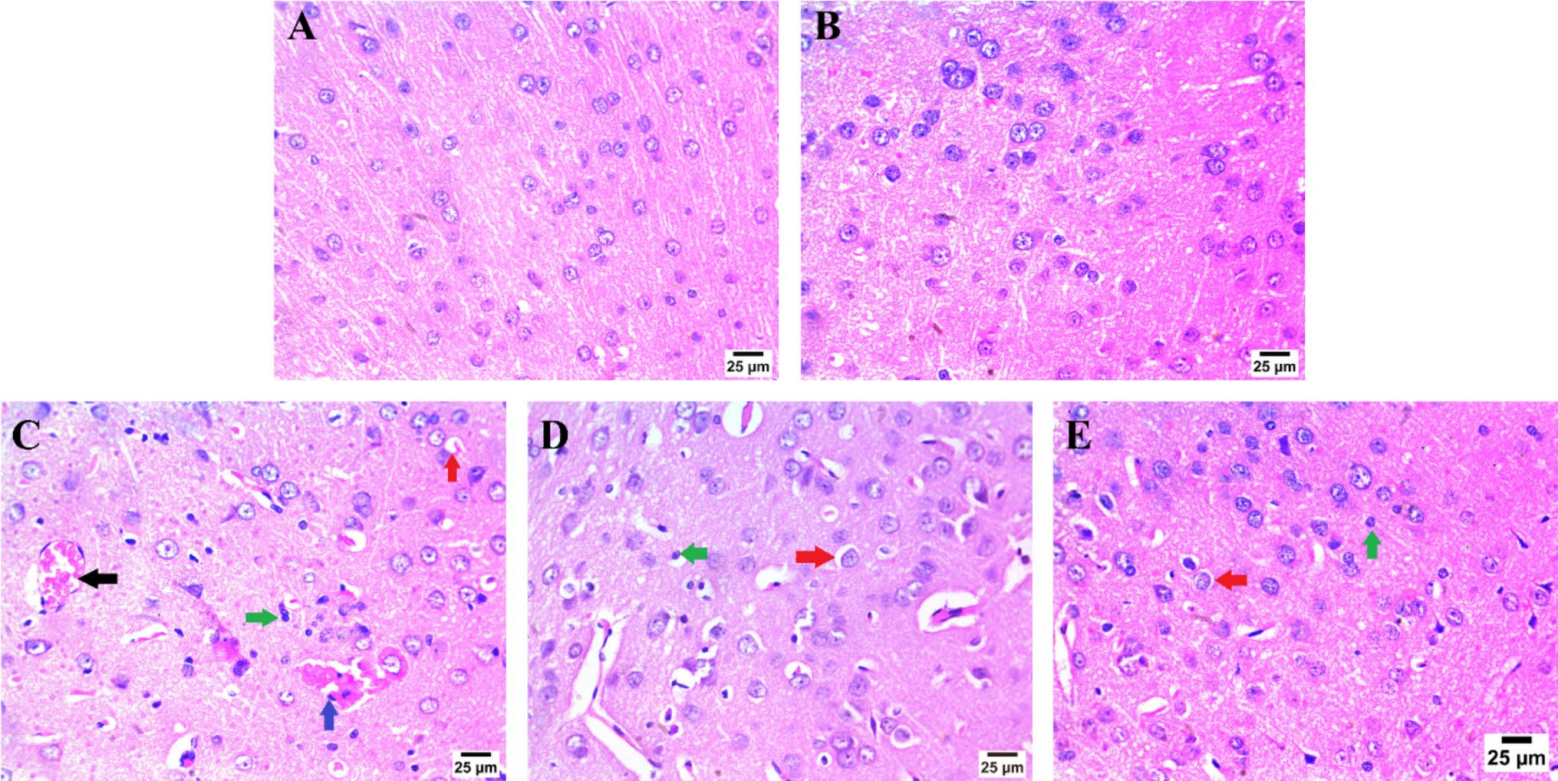
A-J photomicrographs represent staining of striatum with H&E (Scale bar 25 µm). **A** Control group, **B** ART alone treatment, **C** 3-NP group, **D** ART 50 mg treated group, and **E** ART 100 mg treated group. Congestion of minute capillary (black arrow) with hemorrhage (blue arrow), perineuronal edema (red arrow), and severe astrogliosis (green arrow). 3-NP; 3-nitropropionic acid, ART; Artemisinin

Histopathological examination of the striatum in all groups was performed using H&E staining. The striatum exhibited a normal histological structure in both the control and the ART-only treated group. However, the administration of 3-NP resulted in capillary congestion accompanied by hemorrhage, severe perineuronal edema, and pronounced astrogliosis. Interestingly, in the group treated with 50 mg of ART, the striatum showed moderate perineuronal edema with severe astrogliosis. Similarly, cotreatment with 100 mg of ART further improved the condition, resulting in mild perineuronal edema with severe astrogliosis Fig. 2

### Effect of artemisinin on 3-NP-induced changes in striatal GFAP immunoreactivity

**Fig. 3:**
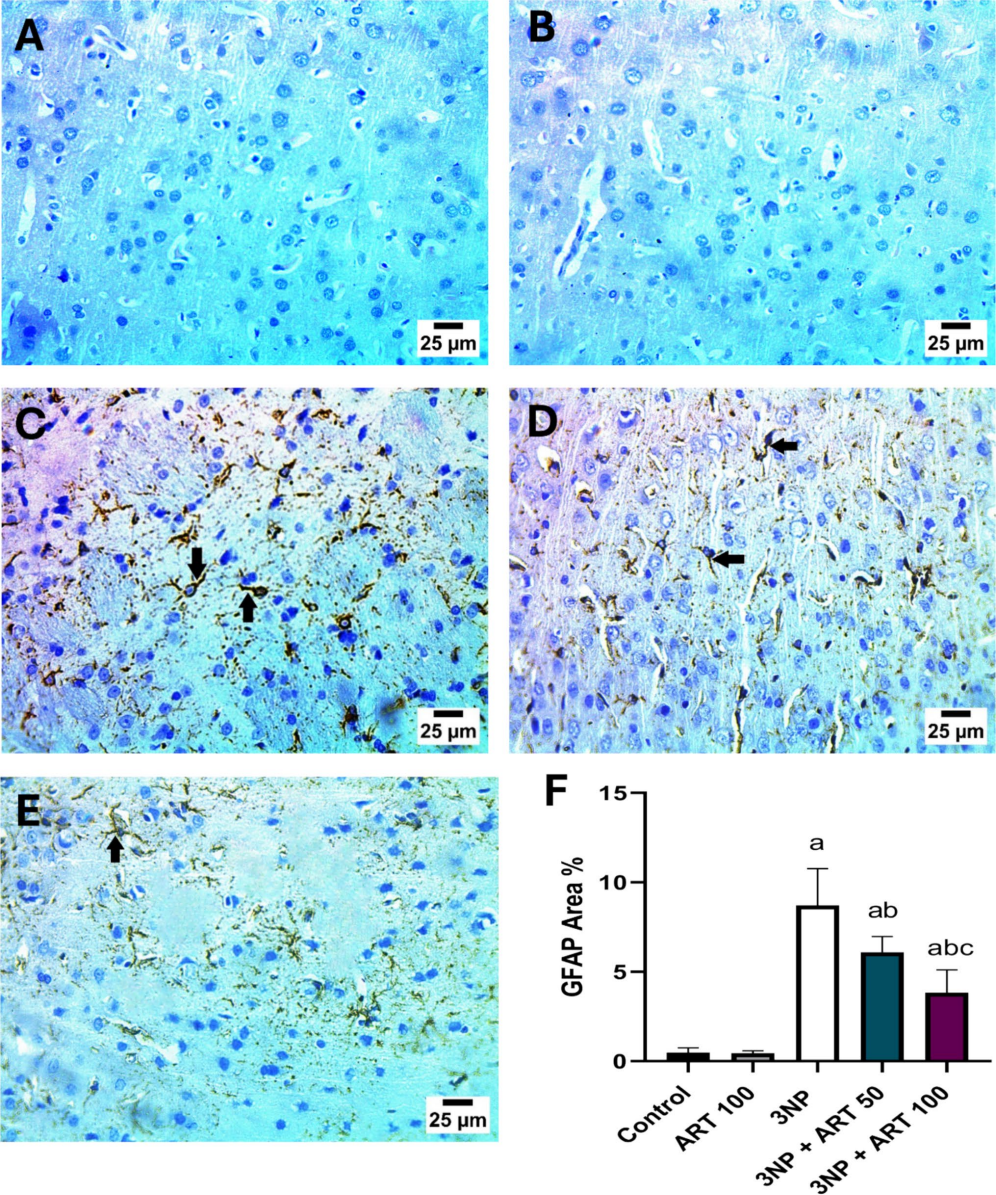
Effect of Artemisinin on 3-NP-induced alteration in striatal GFAP immunoreactivity. **A**–**F** photomicrographs represent immunohistochemical staining of GFAP in the striatum (Scale bar 25 µm). **A** Control group, **B** ART alone treatment, **C** 3-NP group, **D** ART 50 mg treated group, and **E** ART 100 mg treated group. **F** % area of GFAP immunoexpression. Data are presented as mean ± SD. of 3 rats per group, using one-way ANOVA followed by Tukey’s post hoc test; a vs control, b vs 3-NP, c vs 3-NP + ART 50. 3-NP; 3-nitropropionic acid, ART; Artemisinin

Immunoreactivity for striatal GFAP was evaluated by immunostaining to assess the extent of astrocyte activation. Statistical analysis using one-way ANOVA revealed a significant difference between groups (GFAP: F (4, 45) = 97.07, *P* < 0.0001). In the control group and the ART-only group, no detectable GFAP expression was observed in the striatum. However, exposure to 3-NP resulted in a significant increase in GFAP levels (p < 0.0001, 18.24-fold) compared with the control group, indicating strong astrocyte activation. Interestingly, treatment with a low dose of ART (50 mg) significantly reduced GFAP expression by (*p* < 0.0001, 30.13) compared to the 3-NP group. This reduction was even more pronounced with a higher ART dose (100 mg), which significantly decreased GFAP expression by 56.05% (p < 0.0001) compared with the 3-NP group. Notably, the 100 mg ART dose also showed a significant (p = 0.0006, 37.1%) reduction in GFAP levels compared with the 50 mg ART dose group, highlighting the dose-dependent effect of ART in suppressing astrocyte activation Fig. 3

### Effect of artemisinin on striatal contents of TLR4, RIPK1, RIPK3, MLKL, p38 (*p*T180/Y182)-MAPK, HMGB1 in 3-NP rat model

In current study, statistical analysis revealed significant differences among the groups in the levels of TLR4, RIPK1, RIPK3, MLKL, p38 (pT180/Y182)-MAPK, and HMGB1 (TLR4: F (4, 10) = 21.7, *p* < 0.0001), (RIPK1: F (4, 10) = 205.5, *p* < 0.0001), (RIPK3: F (4, 10) = 18.11, *p* = 0.0001), (MLKL: F (4, 10) = 59.84, *p* = 0.0001), (p38 (pT180/Y182)-MAPK: F (4, 10) = 41.7, *p* < 0.0001) and (HMGB1: F (4, 10) = 169.8, *p* < 0.0001), intoxication with 3-NP significantly elevated the levels of TLR4 (*p* < 0.0001, 6.84-fold), RIPK1 (*p* < 0.0001, 6.36-fold), RIPK3 (*p* = 0.0002, 5.2-fold), MLKL (*p* = 0.0001, 5.5-fold), p38MAPK (*p* < 0.0001, 4.22-fold), and HMGB1 (*p* < 0.0001, 4.83-fold) compared to the control group.

**Fig. 4:**
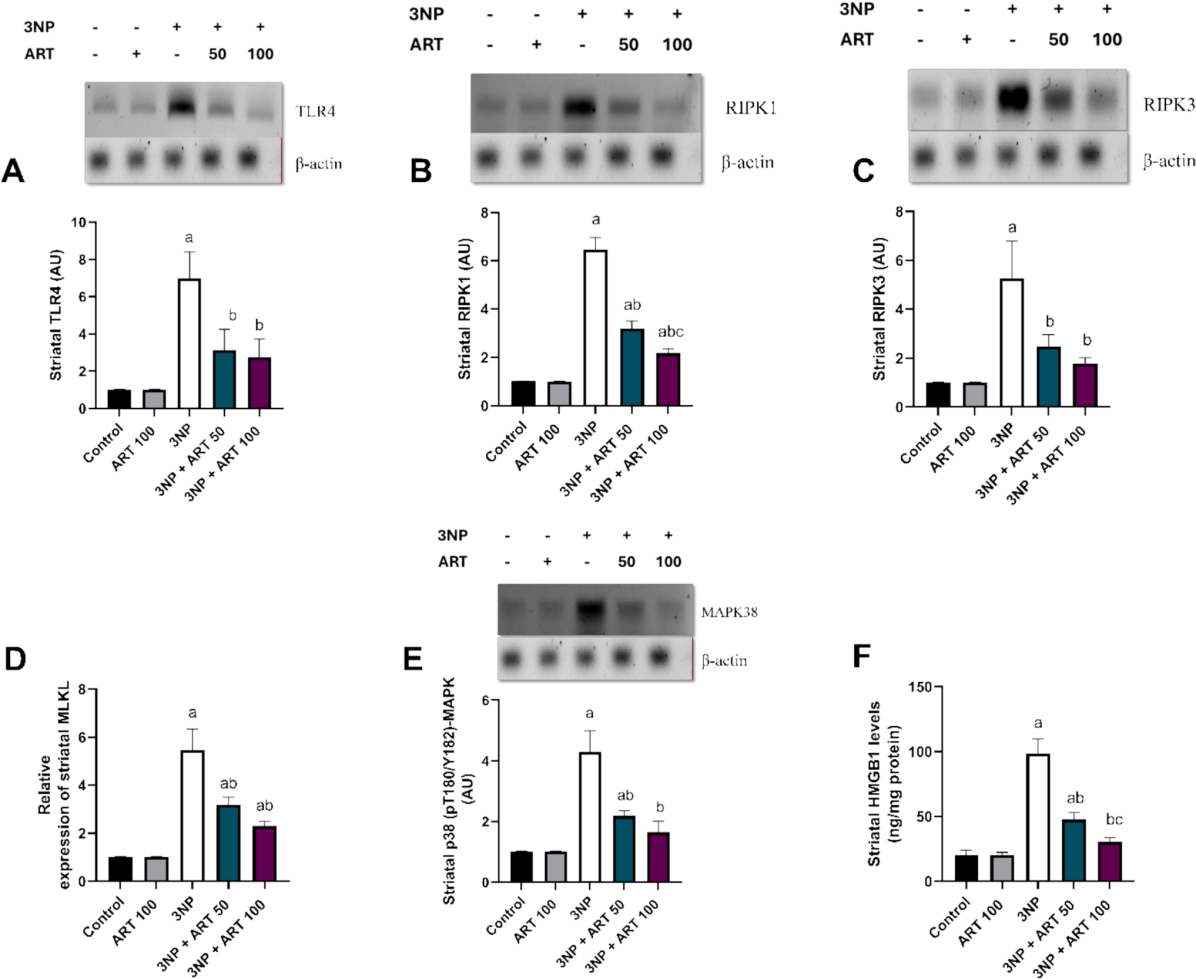
Effect of Artemisinin on 3-NP-induced alteration in the expression of **A** TLR4, **B** RIPK1, **C** RIPK3, **D** MLKL, **E** p38-MAPK, and **F** HMGB1. Data are presented as mean ± SD of 6 mice per group, using one-way ANOVA followed by Tukey’s post hoc test; a vs control, b vs 3-NP, c vs 3-NP + ART 50. 3-NP; 3-nitropropionic acid, ART; Artemisinin

However, treatment with 50 mg of ART significantly reduced the levels of TLR4 (*p* = 0.0029, 55.16%), RIPK1 (*p* < 0.0001, 50.51%), RIPK3 (*p* = 0.0053, 53.06%), MLKL (*p* = 0.0001, 41.6%), p38 (pT180/Y182)-MAPK (*p* = 0.0002, 49.22%), and HMGB1 (*p* < 0.0001, 51.52%) relative to the 3-NP intoxicated group. Similarly, co-administration of 100 mg of ART resulted in a significant decrease in the levels of TLR4 (*p* = 0.0015, 60.39%), RIPK1 (*p* < 0.0001, 66.34%), RIPK3 (*p* = 0.001, 66.41%), MLKL (*p* = 0.0001, 58%), p38 (pT180/Y182)-MAPK (*p* < 0.0001, 61.7%), and HMGB1 (*p* < 0.0001, 69.17%) compared to the 3-NP group. The higher dose of 100 mg ART significantly reduced RIPK1 levels by 31.97% (*p* = 0.0068) and HMGB1 levels by 36.42% (*p* = 0.0004) compared to the 50 mg ART-treated group Fig. 4

### Effect of artemisinin on striatal GSH, Nrf2, MDA, and 8-OHDG in 3-NP rat model

Striatal levels of antioxidants GSH, Nrf2, MDA, and 8-OHdG were analyzed. One-way ANOVA demonstrated significant differences among groups for all markers: GSH (F (4, 25) = 35.65, *p* < 0.0001), Nrf2 (F (4, 25) = 222.3, *p* < 0.0001), MDA (F (4, 25) = 153.3, *p* < 0.0001), and 8-OHdG (F (4, 25) = 287.3, *p* < 0.0001).

3-NP administration significantly decreased GSH and Nrf2 levels by 57.6% and 77.72%, respectively (*p* < 0.0001 vs. control), while markedly increasing MDA and 8-OHdG levels by 4.33 and 4.81-fold (*p* < 0.0001). Treatment with 50 mg ART significantly restored GSH and Nrf2 levels by 1.91-fold and 3.17-fold, respectively (*p* < 0.0001 vs. 3-NP), and reduced MDA and 8-OHdG levels by 45.9% and 64.5% compared to the 3-NP group (*p* < 0.0001).

**Fig. 5:**
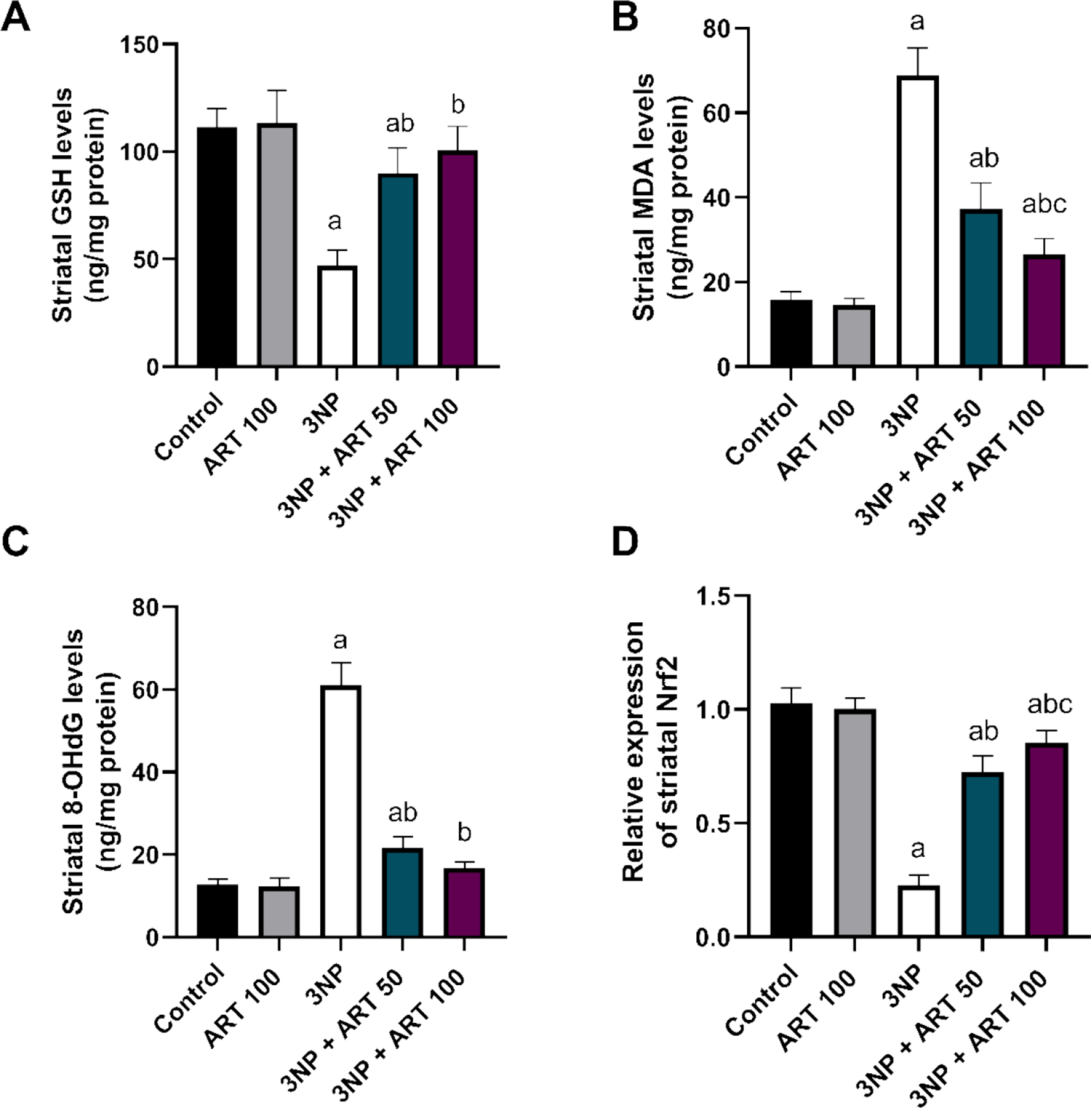
Effect of Artemisinin on 3-NP-induced alteration in **A** GSH, **B** MDA, **C** 8-OHDG level, and **D** Nrf2 expression. Data are presented as mean ± SD of 6 mice per group, using one-way ANOVA followed by Tukey’s post hoc test; a vs control, b vs 3-NP, c vs 3-NP + ART 50. 3-NP; 3-nitropropionic acid, ART; Artemisinin

Co-administration of 100 mg ART significantly elevated GSH and Nrf2 levels by 2.13-fold and 3.73-fold, respectively (*p* < 0.0001 vs. 3-NP), and reduced MDA and 8-OHdG by 61.5% and 72.51% (*p* < 0.0001). Furthermore, the 100 mg dose increased Nrf2 expression by 1.17-fold compared to the 50 mg ART-treated group (*p* < 0.0001) Fig. 5

### Effect of artemisinin on striatal neuroinflammatory markers and SDH in 3-NP rat model

Striatal NF-Κb, TNF-α, and SDH were quantified. One-way ANOVA revealed significant differences among groups for NF-κB (F (4, 25) = 187.3, *p* < 0.0001), TNF-α (F (4, 25) = 174.7, *p* < 0.0001), and SDH (F (4, 25) = 67.42, p < 0.0001).

3-NP intoxication significantly elevated NF-Κb and TNF-α levels by 2.09-fold and 4.52-fold, respectively, while decreasing SDH level by 61.44% (*p* < 0.0001 vs. control). Treatment with 50 mg ART significantly decreased NF-κB by 36.25% and TNF-α by 45.53% and increased SDH to 1.48-fold compared to the 3-NP group (*p* < 0.0001).

**Fig. 6:**
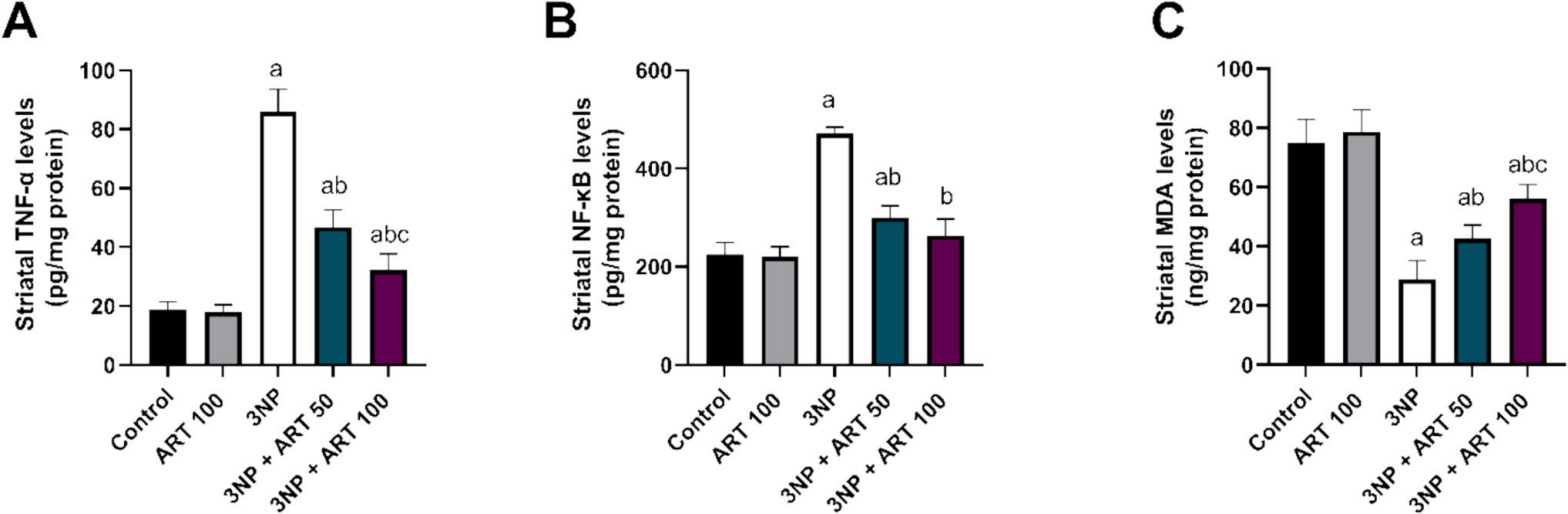
Effect of Artemisinin on 3-NP-induced alteration in **A** TNF-α, **B** NF-κB, and **C** SDH. Data are presented as mean ± SD of 6 mice per group, using one-way ANOVA followed by Tukey’s post hoc test; a vs control, b vs 3-NP, c vs 3-NP + ART 50. 3-NP; 3-nitropropionic acid, ART; Artemisinin

Co-administration of 100 mg ART significantly decreased NF-κB and TNF-α levels by 44.02% and 62.49%, respectively, and increased SDH level to 1.94-fold (*p* < 0.0001 vs. 3-NP). The 100 mg dose also reduced TNF-α by 31.12% compared to the 50 mg ART group (*p* = 0.005) Fig. 6

### Effect of artemisinin on striatal Sig1, D2R, XIAP, and BDNF in 3-NP rat model

Sig1, D2 receptor, XIAP, and BDNF are neuroprotective in various neurodegenerative diseases. In this study, statistical analysis identified significant differences among the experimental groups in the expression levels of these proteins: (Sig1: F (4, 25) = 251.1, *p* < 0.0001), (D2 receptor: F (4, 25) = 222.2, *p* < 0.0001), (XIAP: F (4, 25) = 222.3, *p* < 0.0001), and (BDNF: F (4, 25) = 52.98, *p* < 0.0001).

3-NP exposure significantly decreased Sig1 (86.21%), D2 receptor (64%), XIAP (77.75%), and BDNF (57.61%) levels compared to controls (*p* < 0.0001). Treatment with 50 mg ART significantly restored these markers by 4.57-fold (Sig1), 2.13-fold (D2 receptor), 3.15-fold (XIAP), and 2.18-fold (BDNF) relative to the 3-NP group (*p* < 0.0001).

**Fig. 7:**
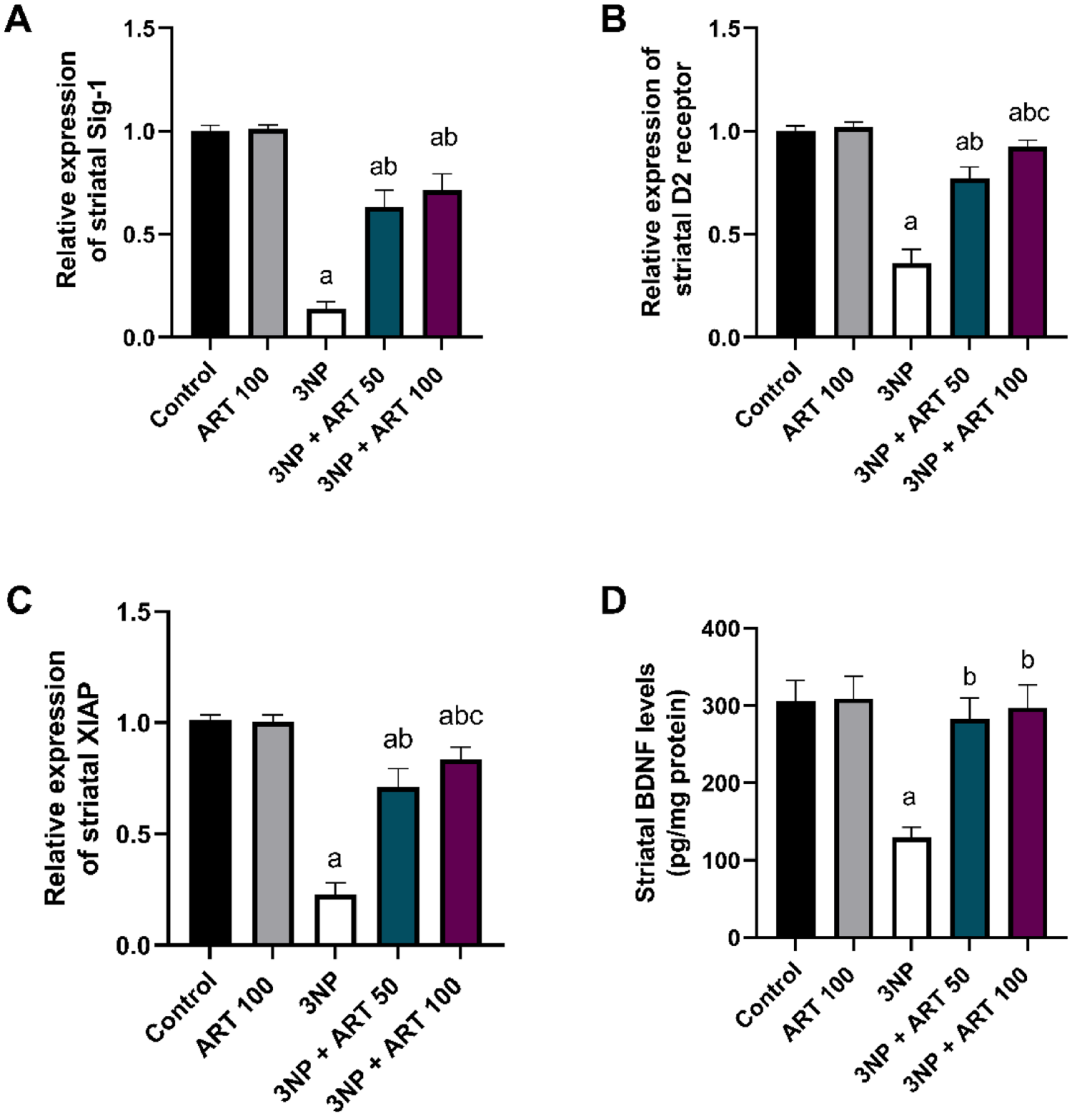
Effect of Artemisinin on 3-NP-induced alteration in **A** Sig1, **B** D2R, **C** XIAP, and **D** BDNF. Data are presented as mean ± SD of 6 mice per group, using one-way ANOVA followed by Tukey’s post hoc test; a vs control, b vs 3-NP, c vs 3-NP + ART 50. 3-NP; 3-nitropropionic acid, ART; Artemisinin

Treatment with 100 mg ART significantly upregulated Sig1 (5.15-fold), D2 receptor (2.56-fold), XIAP (3.71-fold), and BDNF (2.29-fold) compared to the 3-NP group (*p* < 0.0001). Additionally, the 100 mg dose further increased D2 receptor (1.19-fold, *p* < 0.0001) and XIAP (1.17-fold, *p* = 0.003) expression relative to the 50 mg ART-treated group Fig. 7

## Discussion

This work presents initial evidence of the neuroprotective effect of ART against 3-NP-induced neurotoxicity in a rat model. Several lines of evidence substantiated the protective effect: (i) enhancement of motor and cognitive performance, (ii) reduction of oxidative stress, indicated by elevated levels of Nrf2, GSH, and decrease 8-OHdG, (iii) suppression of neuroinflammatory markers, such as NF-κB p65 and TNF-α, (iv) downregulation of HMGB1/TLR4 signaling, in parallel with diminished expression of the downstream RIPK1/RIPK3/p38-MAPK axis, and (v) upregulation of the antiapoptotic protein XIAP and mitochondrial complex II (succinate dehydrogenase, SDH). Collectively, these findings suggest that ART treatment mitigates behavioral, biochemical, and molecular alterations induced by 3-NP (Table 1).

**Table 1:** Primer sequences used for qPCR

mRNA species	Accession number	Primer sequence (5′–3′)
XIAP	BC032729.1	F: AGTGGTAGTCCTGTTTCAGCATCA
		R: CCGCACGGTATCTCCTTCA
Nrf2	NM_006164	F: CAGCGACGGAAAGAGTATGA
		R: TGGGCA ACCTGGGAGTAG
D2R	NM_016574.4	F: CCTTCACCATCTCTTGCC
		R: CTTCTCTGCTGGGAGAGC
Sig1	NM_001286551.1	F: ACCATCATCTCTGGCACCTT
		R: CTCCACCATCCATGTGTTTG
MLKL	NM_152649	F: AGGAGGCTAATGGGGAGATAGA
		R: TGGCTTGCTGTTAGAAACCTG
GAPDH	NM_002046.7	F: GTCTCCTCTGACTTCAACAGCG
		R: ACCACCCTGTTGCTGTAGCCAA

The striatum, a key component of the basal ganglia, is essential for motor coordination, learning, and memory (Jang and Cho 2016). 3-NP, a mitochondrial toxin, has been widely used to create experimental models that mimic the clinical and pathological characteristics of Huntington’s disease. The administration of 3-NP induces selective neurodegeneration, predominantly targeting medium spiny neurons in the striatum, resulting in motor dysfunction, cognitive impairments, and compromised memory retention (Kumar et al. 2007b; Aziz et al. 2008). 3-NP generated hippocampal lesions in the CA1 and CA3 pyramidal neuron areas, closely linked to cognitive function (Kumar and Kumar 2009), and elevated acetylcholinesterase activity, exacerbating cognitive loss. Besides the striatum, 3-NP has been shown to impair several brain areas, including the hippocampus, thalamus, and cerebral cortex (Borlongan et al. 1997). Behavioral research has revealed that mice treated with 3-NP exhibit marked reductions in locomotor activity, compromised coordination, and altered limb reflexes (Kumar et al. 2007a; Bhateja et al. 2012).

Furthermore, histopathological analysis indicates that 3-NP exposure results in significant neuronal degeneration within the lesion core and heterogeneous neuronal reactions in the adjacent transition zone, characterized by pronounced damage to projection neurons. In contrast, interneurons may exhibit proliferative alterations (Mu et al. 2011; Tasset et al. 2011). The neurotoxicity induced by 3-NP is primarily attributed to mitochondrial dysfunction, oxidative stress, disruption of energy metabolism, and the activation of apoptotic pathways, all of which lead to the behavioral and structural deficits seen in human and animal models of Huntington’s disease (Rosenstock et al. 2004; Sandhir and Mehrotra 2013).

This study employed behavioral assays, including the open field, Morris water maze, and novel object recognition tests, to comprehensively evaluate 3-NP-induced motor and cognitive impairments in rats, modeling neurodegenerative deficits characteristic of Huntington’s disease. ART treatment was associated with significant improvement in these behavioral deficits, supporting its potential neuroprotective profile in this experimental context.

Notably, elevated levels of oxidative stress markers, such as malondialdehyde (MDA) and 8-hydroxydeoxyguanosine (8-OHdG), are consistently observed in the brains of patients with Huntington’s disease (Klepac et al. 2007). Postmortem investigations provide robust evidence of its role in HD development. Significantly elevated levels of 8-OHdG have been detected in the striatum and forebrain of R6/2 HD mice and peripheral fluids, including urine and plasma, signifying extensive oxidative DNA damage (Chen 2011). Current data indicate that oxidative stress is intricately associated with disease development and may function as a significant biomarker and therapeutic target in Huntington’s disease.

Moreover, Nuclear factor erythroid two-related factor 2 (Nrf2) is a principal regulator of cellular protection against oxidative stress, inflammation, and mitochondrial dysfunction, which are critical characteristics of neurodegenerative disorders, including HD. Nrf2 governs the expression of various genes implicated in antioxidant responses, detoxification, the clearance of damaged proteins and organelles, and energy metabolism (Luchkova et al. 2024). Its malfunction has been associated with HD and Alzheimer’s disease. Preclinical studies indicate that pharmacological stimulation of Nrf2 improves cellular resilience, postpones disease onset, and reduces neuronal loss. Pre-symptomatic activation of the Nrf2 pathway may mitigate or avert striatal degeneration in HD, rendering it a persuasive treatment approach for many neurodegenerative disorders (Tucci et al. 2022). Recently, Nrf2 has been identified as a novel regulator of necroptosis, and its pharmacological activation was shown to significantly reduce RIP1 and RIP3 expression in ethanol-treated hepatocytes, thereby inhibiting hepatocyte necroptosis (Zhou et al. 2019).

In line with a previous study, ART treatment significantly ameliorated oxidative stress, as indicated by increased Nrf2 levels and decreased 8-OHdG levels (Deng et al. 2025).

In addition to the changes in GSH, Nrf2, and 8-OHdG, our findings also demonstrated significant alterations in MDA levels, a well-established marker of lipid peroxidation. The elevation of MDA in the 3-NP group confirms the contribution of lipid peroxidation to oxidative damage in Huntington’s disease models. Notably, ART treatment markedly reduced MDA levels, further supporting its role as an effective antioxidant and neuroprotective agent.

High-mobility group (HMG) proteins are non-histone DNA-binding proteins ubiquitously expressed in eukaryotic cells and play key roles in diverse biological processes (Dumitriu et al. 2005). Among these, HMGB1 is the most prevalent member, widely distributed across human cells (Ge et al. 2021). In the CNS, HMGB1 is expressed in neurons, microglia, and astrocytes (Manivannan et al. 2021; Huang et al. 2023). When localized on the neuronal cell surface or within the extracellular matrix, HMGB1 facilitates neurite outgrowth, enhances cell migration, and plays a crucial role in neuroinflammatory responses following injury (Wan et al. 2020). During cellular events such as apoptosis, necrosis, and pyroptosis, HMGB1 is actively translocated into the extracellular space (R. Chen et al. 2022b, a). HMGB1 functions as a DAMP in this extracellular milieu, triggering innate immune Activation by recruiting inflammatory and immune cells. Additionally, it stimulates macrophages and endothelial cells to release proinflammatory cytokines, thereby amplifying the inflammatory response (Ren et al. 2023; Li et al. 2024). Furthermore, once immune and endothelial cells are activated by HMGB1, they can subsequently release HMGB1 themselves, perpetuating a self-sustaining positive feedback loop (Kang et al. 2014) in accordance with the previous study that reported that apoptotic and autophagic pathways are activated in the 3-NP model of HD, which is associated with increased expression of HMGB1. This, in turn, causes damage to neurons (Gendy et al. 2023). The present study demonstrated a significant increase in HMGB1, which was attenuated following ART treatment.

Beyond its role in neuroinflammation, HMGB1 is a key mediator of inflammatory responses through its interaction with TLR4 (R. Chen et al. 2022b, a). Its involvement has been implicated in various neurological conditions, including epilepsy and neurodegenerative disorders such as HD (Angelopoulou et al. 2020). Moreover, HMGB1 has been shown to upregulate TNF-α, suggesting its role in apoptosis induction via inflammatory pathways (Song et al. 2021).

TLR4 is a key player in the body’s immune defense system, acting as a pattern recognition receptor (PRR) that detects DAMPs (Kielian 2006; Venegas and Heneka 2017). TLR4 triggers a signaling cascade that activates specific transcription factors, leading to the production of cytokines, chemokines, growth factors, and other inflammatory mediators (Yang et al. 2020). This response is essential for immune defense but can contribute to chronic inflammation and neurodegenerative diseases when dysregulated.

TLR4 signaling follows two major pathways: the MyD88-TNF receptor-associated factor 6 (TRAF6)-TAK1 pathway and the TRIF-TBK1 pathway. The MyD88-TRAF6 pathway is crucial because it activates TAK1, which regulates p38-MAPK, extracellular signal-regulated kinase (ERK), and NF-κB (Iwamoto et al. 2020).

NF-κB plays a central role in inflammation, acting as a master regulator of immune responses. Under normal conditions, it remains inactive in the cytoplasm, bound to its inhibitor, IκB. However, when the immune system detects a threat, IκB is phosphorylated and degraded, freeing NF-κB to enter the nucleus and switch on genes responsible for inflammation (Cui et al. 2010; Napetschnig and Wu 2013; Rodrigues et al. 2022; D. Chen et al. 2022b, a). TLR4 enhances this process by activating the IκB kinase (IKK) complex, which facilitates NF-κB nuclear translocation and amplifies the inflammatory response (Wang et al. 2019).

In addition to NF-κB activation, TLR4 signaling triggers the MAPK pathway, strengthening the inflammatory response by phosphorylating key proteins (Medvedev et al. 2007; Mitchell et al. 2018). This activation induces nuclear translocation of transcription factors, including AP-1, c-Fos, c-Jun, ELK-2, and ATF-2, which collectively enhance proinflammatory cytokine expression (Svensson et al. 2011; Patel et al. 2012; Li et al. 2022). Together, NF-κB and p38 MAPK activation drive the release of inflammatory molecules like TNF-α, IL-1β, IL-6, IL-8, and IL-12, a process particularly prominent in microglia, the brain’s resident immune cells (Landström, 2010; Feng et al. 2014; Mustafa et al. 2024). Interestingly, 3-NP has previously been reported to activate TLR4/NF-κB signaling and to increase p38-MAPK expression (Yang et al. 2021; Gendy et al. 2023). Indeed, 3-NP rats showed marked elevation in the signaling mentioned above. Controversy: ART administration inhibited the 3-NP effect.

Necroptosis is a distinct type of programmed cell death that displays attributes of both apoptosis and necrosis, usually occurring when apoptotic pathways are impaired (Dhuriya and Sharma 2018). In contrast to apoptosis, necroptosis occurs independently of essential apoptotic regulators, including caspases, B-cell lymphoma 2 (Bcl-2), and mitochondrial cytochrome c release (Degterev et al. 2008). This type of cell death serves as an alternative mechanism, especially in apoptosis-resistant contexts, offering a potential benefit for addressing resistance challenges frequently encountered in cancer treatment (Wu et al. 2020). Moreover, neuronal cell death and neuroinflammation in the pathogenesis of certain neurodegenerative diseases may be facilitated by necroptosis. An inflammatory reaction is triggered by the massive release of DAMPs from necroptotic cells (Zhang et al. 2017).

Receptor-interacting protein kinases 1 and 3 (RIPK1 and RIPK3) are crucial in necroptosis (Silke et al. 2015). Several stimuli trigger them, including DAMPs, TNF, IFN-α, TLRs, viral infections, and genotoxic stresses. TNF-α initiates the most well-defined necroptotic pathway (Thapa et al. 2013), leading to the formation of an amyloid-like complex known as the necrosome, composed of RIPK1 and RIPK3 (Cho et al. 2009). The Activation and phosphorylation of RIPK1 commence the necroptosis cascade. Activated RIPK1 subsequently activates its downstream target, RIPK3, which polymerizes and forms a necrosome complex with RIPK1. RIPK3 within the necrosome recruits and phosphorylates its downstream target, including MLKL (Abdel-Magid 2025). These kinases also modulate proinflammatory signaling, which is vital for innate immune protection (Kawai and Akira 2010). A previous study reported that 3-NP induced striatal toxicity via stimulating the necroptosis pathways involving RIPK1, RIPK3, and MLKL signaling in rats (Elbaz et al. 2025). In the current study, ART treatment was associated with attenuation of 3-NP-induced alterations, including modulation of RIPK1 and RIPK3 expression.

Inhibitors of apoptosis (IAP) proteins, including XIAP, IAP1, and IAP2, modulate critical phases of apoptosis by directly inhibiting caspases 3, 7, and 9. These proteins inhibit the amplification of apoptotic signaling mediated by caspase-3. Although IAPs may inhibit apoptosis under various stimuli, their efficacy is diminished in neurodegenerative disorders (Korhonen 2002). For example, XIAP overexpression protects neurons in ischemia and Parkinson’s disease models. In contrast, its degradation has been observed in amyotrophic lateral sclerosis, suggesting that diminished IAP activity may play a significant role in neuronal damage in HD (Goffredo et al. 2005). Herein, 3-NP significantly reduced XIAP expression. At the same time, ART treatment correlated with restoration of XIAP gene expression in striatal tissue.

Brain-derived neurotrophic factor (BDNF), a prominent and well-researched neurotrophin in the mammalian brain, is essential for the functioning of the peripheral and central nervous systems (Mori et al. 2021). Changes in BDNF levels have been suggested as biomarkers for many neurodegenerative diseases. The BDNF/TrkB signaling system is crucial for cerebral development, neuronal plasticity, and functions such as neurogenesis, cellular differentiation, survival, synaptic plasticity, and responses to inflammation, pain, and sensory maturation (Dincheva et al. 2016). Significantly, BDNF has robust neuroprotective and regenerative properties, augmenting neuronal resilience and recovery after damage or degeneration (Pisani et al. 2023). Consistent with previous in vivo studies, 3-NP-treated rats demonstrated neurodegeneration, as evidenced by a marked decrease in BDNF levels (Mustafa et al. 2021). On the other hand, ART showed neuroprotective effects by increasing BDNF levels.

Interestingly, TLR4 activation is also linked to downregulation of the Sigma-1 receptor (Sig1R) via the p38-MAPK and NF-κB pathways. Sig1R is a multifunctional protein that protects brain health (Iwamoto et al. 2020). Sig1R is mainly found at the junction between the endoplasmic reticulum (ER) and mitochondria, an area known as the mitochondria-associated ER membrane (MAM). It helps regulate cellular stress responses, synaptic communication, ion channel function, and gene expression (Nakamura et al. 2019; Schmidt and Kruse 2019). Recent studies suggest that Sig1R agonists can reduce the production of proinflammatory cytokines in microglia, highlighting their potential neuroprotective effects (Wu et al. 2015). Notably, lower levels of Sig1R have been observed in patients with Alzheimer’s disease (AD), Parkinson’s disease (PD), and amyotrophic lateral sclerosis (ALS), further reinforcing its role in neurodegeneration (Hedskog et al. 2013; Prause et al. 2013). Similarly, D2R activation has been associated with neuroprotective effects, including modifying intracellular signaling pathways that enhance neuronal survival. Therefore, the interaction between Sig1R and D2R may collaboratively enhance neuroprotection (Pinton et al. 2015). Herein, ART elevated the level of both Sig1R and D2R, indicating a neuroprotective effect.

Our findings are in agreement with previous reports on other anti-inflammatory and neuroprotective agents investigated in 3-NP models. For instance, curcumin was shown to attenuate 3-NP–induced neurotoxicity by suppressing proinflammatory cytokines and enhancing antioxidant defenses (D’Egidio et al. 2023). Similarly, silymarin improved behavioral and biochemical outcomes by modulating oxidative stress pathways and protecting mitochondria (Haddadi et al. 2024). Melatonin has also been reported to exert protective effects by reducing lipid peroxidation and inhibiting neuroinflammatory signaling (D’Egidio et al. 2023). Consistent with these studies, ART in the present work significantly reduced neuroinflammation, oxidative stress, and necroptotic signaling, thereby reinforcing its potential as a neuroprotective compound against 3-NP-induced striatal injury.

Collectively, 3-NP intoxication led to a noticeable increase in HMGB1/TLR4/p38-MAPK/NF-κB signaling, accompanied by a reduction in Sig1R and D2R levels, in agreement with previous reports. Cotreatment with ART significantly attenuated activation of the HMGB1/TLR4/p38-MAPK/NF-κB axis and restored Sig1R and D2R expression. These findings extend existing evidence on the involvement of this inflammatory signaling pathway in Artemisinin’s biological actions by contextualizing its modulation within a 3-NP-induced Huntington’s disease-like model. Importantly, the observed effects should be interpreted as preclinical and pathway-associated neuroprotective responses in an acute experimental setting, rather than definitive evidence of therapeutic efficacy or CNS-targeted disease modification. Further mechanistic and translational studies will be required to determine the relevance of these pathway associations in chronic or genetic models of Huntington’s disease.

While the present study provides novel insights into the neuroprotective potential of ART against 3-NP-induced Huntington’s disease-like pathology, certain limitations should be acknowledged. First, the treatment period was relatively short, and the employed 3-NP paradigm represents an acute/subacute toxin-based model that may not adequately reflect the long-term therapeutic efficacy of ART or the progressive, genetic nature of Huntington’s disease. Second, although our findings demonstrate modulation of HMGB1/TLR4/NF-κB signaling and attenuation of necroptosis, causal mechanistic verification using genetic knockdown approaches or selective pharmacological inhibitors (e.g., TLR4 antagonists, HMGB1-neutralizing antibodies, or necroptosis inhibitors) was not performed due to the scope of the approved experimental protocol. In addition, pharmacokinetic profiling and direct assessment of BBB penetration were not conducted; therefore, CNS exposure and target engagement of ART under the present dosing regimen were not experimentally confirmed. Moreover, key pathogenic mechanisms implicated in Huntington’s disease, including dysregulation of autophagy, classical apoptotic pathways (e.g., caspase-3 activation and Bax/Bcl-2 balance), and synaptic integrity markers such as PSD-95, were not examined. These constraints limit definitive mechanistic and translational interpretation, and the findings should not be overinterpreted as evidence of established CNS-targeted therapeutic efficacy. Future studies employing prolonged treatment regimens, pathway-specific mechanistic interventions, pharmacokinetic and BBB analyses, and validation in chronic or genetic Huntington’s disease models will be essential to confirm the therapeutic relevance, optimal dosing, bioavailability, and long-term safety of ART before clinical translation can be considered.

## Supplementary Information

Below is the link to the electronic supplementary material.

Supplementary file1 (TIF 5769 KB)

Supplementary file2 (TIF 9336 KB)

Supplementary file3 (TIF 5917 KB)

Supplementary file4 (TIF 7901 KB)

Supplementary file5 (TIF 336 KB)

Supplementary file6 (PDF 527 KB)

## Data Availability

Data will be made available on request.

## References

[CR1] Abdel-Magid, A. F. (2025). Receptor-interacting protein kinase 1 (RIPK1) inhibitors as potential treatment for several inflammatory and neurodegenerative diseases. ACS Med Chem Lett. 10.1021/acsmedchemlett.5c00026 10.1021/acsmedchemlett.5c00026 PMC11831378 39967643

[CR2] Ali H, Dong SXM, Gajanayaka N, Cassol E, Angel JB, Kumar A (2021) Selective induction of cell death in human M1 macrophages by Smac mimetics is mediated by cIAP-2 and RIPK-1/3 through the activation of mTORC. J Immunol 207(9):2359–2373. 10.4049/jimmunol.2100108 34561230 10.4049/jimmunol.2100108

[CR3] Angelopoulou E, Paudel YN, Piperi C (2020) Exploring the role of high-mobility group box 1 (HMGB1) protein in the pathogenesis of Huntington’s disease. J Mol Med 98(3):325–334. 10.1007/s00109-020-01885-z 10.1007/s00109-020-01885-z 32036391

[CR4] Aziz NA, Van Der Burg JM, Landwehrmeyer GB, Brundin P, Stijnen T, EHDI Study Group, Roos RAC (2008) Weight loss in Huntington disease increases with higher CAG repeat number. Neurology 71(19):1506–1513. 10.1212/01.wnl.0000334276.09729.0e 18981372 10.1212/01.wnl.0000334276.09729.0e

[CR5] Bhateja DK, Dhull DK, Gill A, Sidhu A, Sharma S, Reddy BK, Padi SS (2012) Peroxisome proliferator-activated receptor-α activation attenuates 3-nitropropionic acid induced behavioral and biochemical alterations in rats: possible neuroprotective mechanisms. Eur J Pharmacol 674(1):33–43. 10.1016/j.ejphar.2011.10.029 22056833 10.1016/j.ejphar.2011.10.029

[CR6] Borlongan CV, Koutouzis TK, Freeman TB, Hauser RA, Cahill DW, Sanberg PR (1997) Hyperactivity and hypoactivity in a rat model of Huntington’s disease: the systemic 3-nitropropionic acid model. Brain Res Protoc 1(3):253–257. 10.1016/s1385-299x(96)00037-2 10.1016/s1385-299x(96)00037-2 9385062

[CR7] Chen C-M (2011) Mitochondrial dysfunction, metabolic deficits, and increased oxidative stress in Huntington’s disease. Chang Gung Med J 34(2):135–152 21539755

[CR8] Chen J, Xu ZC, Xu XM, Zhang JH (2019) Animal models of acute neurological injury. Springer

[CR9] Chen D, Kang H, Tuo T, Wang L, Xia Y, Zhang Y, Zhou L, Ge X, Han J, Guo X (2022a) Astragalus polysaccharide alleviated the inhibition of CSFV C-strain replication caused by PRRSV via the TLRs/NF‑κB/TNF-α pathways. Virus Res 319:198854. 10.1016/j.virusres.2022.198854 35788015 10.1016/j.virusres.2022.198854

[CR10] Chen R, Kang R, Tang D (2022b) The mechanism of HMGB1 secretion and release. Exp Mol Med 54(2):91–102. 10.1038/s12276-022-00736-w 35217834 10.1038/s12276-022-00736-w PMC8894452

[CR11] Cho I-H (2012) Effects of Panax ginseng in neurodegenerative diseases. J Ginseng Res 36(4):342. 10.5142/jgr.2012.36.4.342 23717136 10.5142/jgr.2012.36.4.342 PMC3659610

[CR12] Cho Y, Challa S, Moquin D, Genga R, Ray TD, Guildford M, Chan FK-M (2009) Phosphorylation-driven assembly of the RIP1-RIP3 complex regulates programmed necrosis and virus-induced inflammation. Cell 137(6):1112–1123. 10.1016/j.cell.2009.05.037 19524513 10.1016/j.cell.2009.05.037 PMC2727676

[CR13] Cui J, Zhu L, Xia X, Wang HY, Legras X, Hong J, Ji J, Shen P, Zheng S, Chen ZJ (2010) NLRC5 negatively regulates the NF-κB and type I interferon signaling pathways. Cell 141(3):483–496. 10.1016/j.cell.2010.03.040 20434986 10.1016/j.cell.2010.03.040 PMC3150216

[CR14] D’Egidio F, Castelli V, Cimini A, d’Angelo M (2023) Cell rearrangement and oxidant/antioxidant imbalance in Huntington’s disease. Antioxidants 12(3):571. 10.3390/antiox12030571 36978821 10.3390/antiox12030571 PMC10045781

[CR15] Degterev A, Hitomi J, Germscheid M, Ch’en IL, Korkina O, Teng X, Abbott D, Cuny GD, Yuan C, Wagner G (2008) Identification of RIP1 kinase as a specific cellular target of necrostatins. Nat Chem Biol 4(5):313–321. 10.1038/nchembio.83 18408713 10.1038/nchembio.83 PMC5434866

[CR16] Deng P-x, Silva M, Yang N, Wang Q, Meng X, Ye K-q, Gao H-c, Zheng W-h (2025) Artemisinin inhibits neuronal ferroptosis in Alzheimer’s disease models by targeting KEAP1. Acta Pharmacol Sin 46(2):326–337. 10.1038/s41401-024-01378-6 39251858 10.1038/s41401-024-01378-6 PMC11747332

[CR17] Dhuriya YK, Sharma D (2018) Necroptosis: a regulated inflammatory mode of cell death. J Neuroinflammation 15:1–9. 10.1186/s12974-018-1235-0 29980212 10.1186/s12974-018-1235-0 PMC6035417

[CR18] Dincheva I, Lynch NB, Lee FS (2016) The role of BDNF in the development of fear learning. Depress Anxiety 33(10):907–916. 10.1002/da.22497 27699937 10.1002/da.22497 PMC5089164

[CR19] Dumitriu IE, Baruah P, Manfredi AA, Bianchi ME, Rovere-Querini P (2005) HMGB1: guiding immunity from within. Trends Immunol 26(7):381–387. 10.1016/j.it.2005.04.009 15978523 10.1016/j.it.2005.04.009

[CR20] Elbaz EM, Sayed RH, Abdelkader AA, Fahim AT (2025) Neuroprotective role of morin hydrate on 3-nitropropionic acid-elicited huntington’s disease: in vivo investigation of RIPK1/RIPK3/MLKL necroptosis signaling pathway. Mol Med 31(1):135. 10.1186/s10020-025-01172-y 40217493 10.1186/s10020-025-01172-y PMC11987198

[CR21] Feng X, Wu C-Y, Burton F, Loh HH, Wei L-N (2014) β-arrestin protects neurons by mediating endogenous opioid arrest of inflammatory microglia. Cell Death Differ 21(3):397–406. 10.1038/cdd.2013.152 24162663 10.1038/cdd.2013.152 PMC3921587

[CR22] Gao Y, Chu S-f, Li J-p, Zhang Z, Yan J-q, Wen Z-l, Xia C-y, Mou Z, Wang Z-z, He W-b (2015) Protopanaxtriol protects against 3-nitropropionic acid-induced oxidative stress in a rat model of Huntington’s disease. Acta Pharmacol Sin 36(3):311–322. 10.1038/aps.2014.107 25640478 10.1038/aps.2014.107 PMC4349920

[CR23] Ge Y, Huang M, Yao Y-m (2021) The effect and regulatory mechanism of high mobility group box-1 protein on immune cells in inflammatory diseases. Cells 10(5):1044. 10.3390/cells10051044 33925132 10.3390/cells10051044 PMC8145631

[CR24] Gendy AM, El-Sadek HM, Amin MM, Ahmed KA, El-Sayed MK, El-Haddad AE, Soubh A (2023) Glycyrrhizin prevents 3-nitropropionic acid-induced neurotoxicity by downregulating HMGB1/TLR4/NF-κB p65 signaling, and attenuating oxidative stress, inflammation, and apoptosis in rats. Life Sci 314:121317. 10.1016/j.lfs.2022.121317 36566881 10.1016/j.lfs.2022.121317

[CR25] Goffredo D, Rigamonti D, Zuccato C, Tartari M, Valenza M, Cattaneo E (2005) Prevention of cytosolic IAPs degradation: a potential pharmacological target in Huntington’s Disease. Pharmacol Res 52(2):140–150. 10.1016/j.phrs.2005.01.006 15967379 10.1016/j.phrs.2005.01.006

[CR26] Haddadi R, Eyvari-Brooshghalan S, Makhdoomi S, Fadaiie A, Komaki A, Daneshvar A (2024) Neuroprotective effects of silymarin in 3-nitropropionic acid-induced neurotoxicity in male mice: improving behavioral deficits by attenuating oxidative stress and neuroinflammation. Naunyn-Schmiedebergs Arch Pharmacol 397(4):2447–2463. 10.1007/s00210-023-02776-z 37847410 10.1007/s00210-023-02776-z

[CR27] Hedskog L, Pinho CM, Filadi R, Rönnbäck A, Hertwig L, Wiehager B, Larssen P, Gellhaar S, Sandebring A, Westerlund M (2013) Modulation of the endoplasmic reticulum–mitochondria interface in Alzheimer’s disease and related models. Proc Natl Acad Sci USA 110(19):7916–7921. 10.1073/pnas.1300677110 23620518 10.1073/pnas.1300677110 PMC3651455

[CR28] Huang X, Wang B, Yang J, Lian Y-J, Yu H-Z, Wang Y-X (2023) HMGB1 in depression: an overview of microglial HMBG1 in the pathogenesis of depression. Brain Behavior Immunity-Health 30:100641. 10.1016/j.bbih.2023.100641 37288063 10.1016/j.bbih.2023.100641 PMC10242493

[CR29] Ibrahim WW, Abdel Rasheed NO (2022) Diapocynin neuroprotective effects in 3-nitropropionic acid Huntington’s disease model in rats: emphasis on Sirt1/Nrf2 signaling pathway. Inflammopharmacology 30(5):1745–1758. 10.1007/s10787-022-01004-z 35639233 10.1007/s10787-022-01004-z PMC9499906

[CR30] Iwamoto M, Nakamura Y, Takemura M, Hisaoka-Nakashima K, Morioka N (2020) TLR4-TAK1-p38 MAPK pathway and HDAC6 regulate the expression of sigma-1 receptors in rat primary cultured microglia. J Pharmacol Sci 144(1):23–29. 10.1016/j.jphs.2020.06.007 32653342 10.1016/j.jphs.2020.06.007

[CR31] Jang M, Cho I-H (2016) Sulforaphane ameliorates 3-nitropropionic acid-induced striatal toxicity by activating the Keap1-Nrf2-ARE pathway and inhibiting the MAPKs and NF-κB pathways. Mol Neurobiol 53(4):2619–2635. 10.1007/s12035-015-9230-2 26096705 10.1007/s12035-015-9230-2

[CR32] Kang R, Chen R, Zhang Q, Hou W, Wu S, Cao L, Huang J, Yu Y, Fan X-g, Yan Z (2014) HMGB1 in health and disease. Mol Aspects Med 40:1–116. 10.1016/j.mam.2014.05.001 25010388 10.1016/j.mam.2014.05.001 PMC4254084

[CR33] Kawai T, Akira S (2010) The role of pattern-recognition receptors in innate immunity: update on Toll-like receptors. Nat Immunol 11(5):373–384. 10.1038/ni.1863 20404851 10.1038/ni.1863

[CR34] Kielian T (2006) Toll-like receptors in central nervous system glial inflammation and homeostasis. J Neurosci Res 83(5):711–730. 10.1038/ni.1863 16541438 10.1002/jnr.20767 PMC2440498

[CR35] Klepac N, Relja M, Klepac R, Hećimović S, Babić T, Trkulja V (2007) Oxidative stress parameters in plasma of Huntington’s disease patients, asymptomatic Huntington’s disease gene carriers and healthy subjects: a cross-sectional study. J Neurol 254:1676–1683. 10.1007/s00415-007-0611-y 17990062 10.1007/s00415-007-0611-y

[CR36] Korhonen L (2002) *Antiapoptotic proteins in nerve cell survival and neurodegeneration* Acta Universitatis Upsaliensis

[CR37] Kumar P, Kumar A (2009) Neuroprotective effect of cyclosporine and FK506 against 3-nitropropionic acid induced cognitive dysfunction and glutathione redox in rat: possible role of nitric oxide. Neurosci Res 63(4):302–314. 10.1016/j.neures.2009.01.005 19367792 10.1016/j.neures.2009.01.005

[CR38] Kumar P, Padi S, Naidu P, Kumar A (2007a) Cyclooxygenase inhibition attenuates 3-nitropropionic acid-induced neurotoxicity in rats: possible antioxidant mechanisms. Fundam Clin Pharmacol 21(3):297–306. 10.1111/j.1472-8206.2007.00485.x 17521299 10.1111/j.1472-8206.2007.00485.x

[CR39] Kumar P, Padi S, Naidu P, Kumar A (2007b) Possible neuroprotective mechanisms of curcumin in attenuating 3-nitropropionic acid-induced neurotoxicity. Methods Find Exp Clin Pharmacol 29(1):19–26. 10.1358/mf.2007.29.1.1063492 17344940 10.1358/mf.2007.29.1.1063492

[CR40] Kumar P, Kalonia H, Kumar A (2010) Huntington’s disease: pathogenesis to animal models. Pharmacol Rep 62(1):1–14. 10.1016/s1734-1140(10)70238-3 20360611 10.1016/s1734-1140(10)70238-3

[CR41] Landström M (2010) The TAK1–TRAF6 signalling pathway. Int J Biochem Cell Biol 42(5):585–589. 10.1016/j.biocel.2009.12.023 20060931 10.1016/j.biocel.2009.12.023

[CR42] Lawlor KE, Khan N, Mildenhall A, Gerlic M, Croker BA, D’Cruz AA, Hall C, Kaur Spall S, Anderton H, Masters SL, Rashidi M, Wicks IP, Alexander WS, Mitsuuchi Y, Benetatos CA, Condon SM, Wong WW, Silke J, Vaux DL, Vince JE (2015) RIPK3 promotes cell death and NLRP3 inflammasome activation in the absence of MLKL. Nat Commun 6:6282. 10.1038/ncomms7282 25693118 10.1038/ncomms7282 PMC4346630

[CR43] Li X, Xu M, Shen J, Li Y, Lin S, Zhu M, Pang Q, Tan X, Tang J (2022) Sorafenib inhibits LPS-induced inflammation by regulating Lyn-MAPK-NF-kB/AP-1 pathway and TLR4 expression. Cell Death Discov 8(1):281. 10.1038/s41420-022-01073-7 35680841 10.1038/s41420-022-01073-7 PMC9184561

[CR44] Li J, Wang Z, Li J, Zhao H, Ma Q (2024) HMGB1: a new target for ischemic stroke and hemorrhagic transformation. Transl Stroke Res 16(3):990–1015. 10.1007/s12975-024-01258-5 38740617 10.1007/s12975-024-01258-5 PMC12045843

[CR45] Lin SP, Wei JX, Hu JS, Bu JY, Zhu LD, Li Q, Liao HJ, Lin PY, Ye S, Chen SQ, Chen XH (2021) Artemisinin improves neurocognitive deficits associated with sepsis by activating the AMPK axis in microglia. Acta Pharmacol Sin 42(7):1069–1079. 10.1038/s41401-021-00634-3 33758353 10.1038/s41401-021-00634-3 PMC8209200

[CR46] Luchkova A, Mata A, Cadenas S (2024) Nrf2 as a regulator of energy metabolism and mitochondrial function. FEBS Lett 598(17):2092–2105. 10.1002/1873-3468.14993 39118293 10.1002/1873-3468.14993

[CR47] Manivannan S, Wales E, Zaben M (2021) The role of hmgb1 in traumatic brain injury—bridging the gap between the laboratory and clinical studies. Curr Neurol Neurosci Rep 21(12):1–7. 10.1007/s11910-021-01158-3 10.1007/s11910-021-01158-3 34870759

[CR48] Medvedev AE, Piao W, Shoenfelt J, Rhee SH, Chen H, Basu S, Wahl LM, Fenton MJ, Vogel SN (2007) Role of TLR4 tyrosine phosphorylation in signal transduction and endotoxin tolerance. J Biol Chem 282(22):16042–16053. 10.1074/jbc.M606781200 17392283 10.1074/jbc.M606781200 PMC2675888

[CR49] Mitchell J, Kim SJ, Seelmann A, Veit B, Shepard B, Im E, Rhee SH (2018) Src family kinase tyrosine phosphorylates Toll-like receptor 4 to dissociate MyD88 and Mal/Tirap, suppressing LPS-induced inflammatory responses. Biochem Pharmacol 147:119–127. 10.1016/j.bcp.2017.11.015 29175418 10.1016/j.bcp.2017.11.015 PMC5733702

[CR50] Mori Y, Tsuji M, Oguchi T, Kasuga K, Kimura A, Futamura A, Sugimoto A, Kasai H, Kuroda T, Yano S (2021) Serum BDNF as a potential biomarker of Alzheimer’s disease: verification through assessment of serum, cerebrospinal fluid, and medial temporal lobe atrophy. Front Neurol 12:653267. 10.3389/fneur.2021.653267 33967943 10.3389/fneur.2021.653267 PMC8102980

[CR51] Mu S, OuYang L, Liu B, Zhu Y, Li K, Zhan M, Liu Z, Jia Y, Lei W, Reiner A (2011) Preferential interneuron survival in the transition zone of 3‐NP‐induced striatal injury in rats. J Neurosci Res 89(5):744–754. 10.1002/jnr.22591 21337370 10.1002/jnr.22591

[CR52] Mustafa AM, Rabie MA, Zaki HF, Shaheen AM (2021) Inhibition of brain GTP cyclohydrolase I attenuates 3-nitropropionic acid-induced striatal toxicity: involvement of Mas receptor/PI3k/Akt/CREB/BDNF axis. Front Pharmacol 12:740966. 10.3389/fphar.2021.740966 35002694 10.3389/fphar.2021.740966 PMC8727546

[CR53] Mustafa AM, Shaheen AM, Zaki HF, Rabie MA (2024) Nicorandil and carvedilol mitigates motor deficits in experimental autoimmune encephalomyelitis-induced multiple sclerosis: role of TLR4/TRAF6/MAPK/NF-κB signalling cascade. Int Immunopharmacol 127:111387. 10.1016/j.intimp.2023.111387 38134593 10.1016/j.intimp.2023.111387

[CR54] Nakamura Y, Dryanovski DI, Kimura Y, Jackson SN, Woods AS, Yasui Y, Tsai S-Y, Patel S, Covey DP, Su T-P (2019) Cocaine-induced endocannabinoid signaling mediated by sigma-1 receptors and extracellular vesicle secretion. Elife 8:e47209. 10.7554/eLife.47209 31596232 10.7554/eLife.47209 PMC6850780

[CR55] Napetschnig J, Wu H (2013) Molecular basis of NF-κB signaling. Annu Rev Biophys 42(1):443–468. 10.1146/annurev-biophys-083012-130338 23495970 10.1146/annurev-biophys-083012-130338 PMC3678348

[CR56] Patel H, Shaw SG, Shi-Wen X, Abraham D, Baker DM, Tsui JC (2012) Toll‐like receptors in ischaemia and its potential role in the pathophysiology of muscle damage in critical limb ischaemia. Cardiol Res Pract 2012(1):121237. 10.1155/2012/121237 22454775 10.1155/2012/121237 PMC3290818

[CR57] Pinton L, O Borroto-Escuela D, Narváez M, Oflijan J, F Agnati L, Fuxe K (2015) Evidence for the existence of dopamine D2R and Sigma 1 allosteric receptor-receptor interaction in the rat brain: role in brain plasticity and cocaine action. Springerplus 4:1–3225674489

[CR58] Pisani A, Paciello F, Del Vecchio V, Malesci R, De Corso E, Cantone E, Fetoni AR (2023) The role of BDNF as a biomarker in cognitive and sensory neurodegeneration. J Pers Med 13(4):652. 10.3390/jpm13040652 37109038 10.3390/jpm13040652 PMC10140880

[CR59] Prause J, Goswami A, Katona I, Roos A, Schnizler M, Bushuven E, Dreier A, Buchkremer S, Johann S, Beyer C (2013) Altered localization, abnormal modification and loss of function of Sigma receptor-1 in amyotrophic lateral sclerosis. Hum Mol Genet 22(8):1581–1600. 10.1093/hmg/ddt008 23314020 10.1093/hmg/ddt008

[CR60] Ramachandran S, Thangarajan S (2018) Thymoquinone loaded solid lipid nanoparticles counteracts 3-Nitropropionic acid induced motor impairments and neuroinflammation in rat model of Huntington’s disease. Metab Brain Dis 33(5):1459–1470. 10.1007/s11011-018-0252-0 29855977 10.1007/s11011-018-0252-0

[CR61] Ren W, Zhao L, Sun Y, Wang X, Shi X (2023) HMGB1 and Toll-like receptors: potential therapeutic targets in autoimmune diseases. Mol Med 29(1):117. 10.1186/s10020-023-00717-3 37667233 10.1186/s10020-023-00717-3 PMC10478470

[CR62] Rodrigues M, Petrova T, Tibbs B, Arthur JSC, Cohen P (2022) TAK1 protein kinase activity is required for TLR signalling and cytokine production in myeloid cells. Biochem J 479(17):1891–1907. 10.1042/BCJ20220314 36062803 10.1042/BCJ20220314 PMC9555797

[CR63] Rosenstock T, Carvalho A, Jurkiewicz A, Frussa‐Filho R, Smaili S (2004) Mitochondrial calcium, oxidative stress and apoptosis in a neurodegenerative disease model induced by 3‐nitropropionic acid. J Neurochem 88(5):1220–1228. 10.1046/j.1471-4159.2003.02250.x 15009678 10.1046/j.1471-4159.2003.02250.x

[CR64] Sandhir R, Mehrotra A (2013) Quercetin supplementation is effective in improving mitochondrial dysfunctions induced by 3-nitropropionic acid: implications in Huntington’s disease. Biochimica Et Biophysica Acta (BBA) 1832(3):421–430. 10.1016/j.bbadis.2012.11.018 23220257 10.1016/j.bbadis.2012.11.018

[CR65] Sayed NH, Fathy N, Kortam MA, Rabie MA, Mohamed AF, Kamel AS (2020) Vildagliptin attenuates Huntington’s disease through activation of GLP-1 receptor/PI3K/Akt/BDNF pathway in 3-nitropropionic acid rat model. Neurotherapeutics 17(1):252–268. 10.1007/s13311-019-00805-5 31728850 10.1007/s13311-019-00805-5 PMC7007456

[CR66] Schmidt HR, Kruse AC (2019) The molecular function of σ receptors: past, present, and future. Trends Pharmacol Sci 40(9):636–654. 10.1016/j.tips.2019.07.006 31387763 10.1016/j.tips.2019.07.006 PMC6748033

[CR67] Sidhu A, Diwan V, Kaur H, Bhateja D, Singh CK, Sharma S, Padi SS (2018) Nicotinamide reverses behavioral impairments and provides neuroprotection in 3˗ nitropropionic acid induced animal model of Huntington’s disease: implication of oxidative stress˗ poly (ADP˗ ribose) polymerase pathway. Metab Brain Dis 33:1911–1921. 10.1007/s11011-018-0297-0 30054774 10.1007/s11011-018-0297-0

[CR68] Silke J, Rickard JA, Gerlic M (2015) The diverse role of RIP kinases in necroptosis and inflammation. Nat Immunol 16(7):689–697. 10.1038/ni.3206 26086143 10.1038/ni.3206

[CR69] Song H-H, Song T-C, Yang T, Sun C-S, He B-Q, Li H, Wang Y-J, Li Y, Wu H, Hu Y-M (2021) High mobility group box 1 mediates inflammatory response of astrocytes via cyclooxygenase 2/prostaglandin E2 signaling following spinal cord injury. Neural Regen Res 16(9):1848–1855. 10.4103/1673-5374.303039 33510092 10.4103/1673-5374.303039 PMC8328776

[CR70] Suganya SN, Sumathi T (2017) Effect of rutin against a mitochondrial toxin, 3-nitropropionicacid induced biochemical, behavioral and histological alterations-a pilot study on Huntington’s disease model in rats. Metab Brain Dis 32:471–481. 10.1007/s11011-016-9929-4 27928694 10.1007/s11011-016-9929-4

[CR71] Svensson C, Part K, Künnis-Beres K, Kaldmäe M, Fernaeus SZ, Land T (2011) Pro-survival effects of JNK and p38 MAPK pathways in LPS-induced activation of BV-2 cells. Biochem Biophys Res Commun 406(3):488–492. 10.1016/j.bbrc.2011.02.083 21338578 10.1016/j.bbrc.2011.02.083

[CR72] Tasset I, Agüera E, Olmo-Camacho R, Escribano B, Sánchez-López F, Delgado MJ, Cruz AH, Gascón F, Luque E, Peña J (2011) Melatonin improves 3-nitropropionic acid induced behavioral alterations and neurotrophic factors levels. Prog Neuro-Psychopharmacol Biol Psychiatry 35(8):1944–1949. 10.1016/j.pnpbp.2011.09.005 10.1016/j.pnpbp.2011.09.005 21939726

[CR73] Thapa RJ, Nogusa S, Chen P, Maki JL, Lerro A, Andrake M, Rall GF, Degterev A, Balachandran S (2013) Interferon-induced RIP1/RIP3-mediated necrosis requires PKR and is licensed by FADD and caspases. Proc Natl Acad Sci USA 110(33):E3109–E3118. 10.1073/pnas.1301218110 23898178 10.1073/pnas.1301218110 PMC3746924

[CR74] Tucci P, Lattanzi R, Severini C, Saso L (2022) Nrf2 pathway in Huntington’s disease (HD): what is its role? Int J Mol Sci. 10.3390/ijms232315272 36499596 10.3390/ijms232315272 PMC9739588

[CR75] Venegas C, Heneka MT (2017) Danger-associated molecular patterns in Alzheimer’s disease. J Leukoc Biol 101(1):87–98. 10.1189/jlb.3MR0416-204R 28049142 10.1189/jlb.3MR0416-204R

[CR76] Wan G, An Y, Tao J, Wang Y, Zhou Q, Yang R, Liang Q (2020) MicroRNA-129-5p alleviates spinal cord injury in mice via suppressing the apoptosis and inflammatory response through HMGB1/TLR4/NF-κB pathway. Biosci Rep 40(3):BSR20193315. 10.1042/BSR20193315 32096822 10.1042/BSR20193315 PMC7069919

[CR77] Wang Y, Hu H, Yin J, Shi Y, Tan J, Zheng L, Wang C, Li X, Xue M, Liu J (2019) TLR4 participates in sympathetic hyperactivity Post-MI in the PVN by regulating NF-κB pathway and ROS production. Redox Biol 24:101186. 10.1016/j.redox.2019.101186 30978539 10.1016/j.redox.2019.101186 PMC6460304

[CR78] Wei H, Wu C, Yuan Y, Lai L (2023) Uncovering the Achilles heel of genetic heterogeneity: Machine learning-based classification and immunological properties of necroptosis clusters in Alzheimer’s disease. Front Aging Neurosci 15:1249682. 10.3389/fnagi.2023.1249682 37799623 10.3389/fnagi.2023.1249682 PMC10548137

[CR79] Wu CL, Hwang CS, Chen SD, Yin JH, Yang DI (2010) Neuroprotective mechanisms of brain-derived neurotrophic factor against 3-nitropropionic acid toxicity: therapeutic implications for Huntington’s disease. Ann NY Acad Sci 1201(1):8–12. 10.1111/j.1749-6632.2010.05628.x 20649532 10.1111/j.1749-6632.2010.05628.x

[CR80] Wu Z, Li L, Zheng LT, Xu Z, Guo L, Zhen X (2015) Allosteric modulation of sigma-1 receptors by SKF 83959 inhibits microglia-mediated inflammation. J Neurochem 134(5):904–914. 10.1111/jnc.13182 26031312 10.1111/jnc.13182

[CR81] Wu Y, Dong G, Sheng C (2020) Targeting necroptosis in anticancer therapy: mechanisms and modulators. Acta Pharm Sin B 10(9):1601–1618. 10.1016/j.apsb.2020.01.007 33088682 10.1016/j.apsb.2020.01.007 PMC7563021

[CR82] Xu G, Huang Y-L, Li P-L, Guo H-M, Han X-P (2017a) Neuroprotective effects of Artemisinin against isoflurane-induced cognitive impairments and neuronal cell death involve JNK/ERK1/2 signalling and improved hippocampal histone acetylation in neonatal rats. J Pharm Pharmacol 69(6):684–697. 10.1111/jphp.12704 28294340 10.1111/jphp.12704

[CR83] Xu G, Huang YL, Li PL, Guo HM, Han XP (2017b) Neuroprotective effects of artemisinin against isoflurane-induced cognitive impairments and neuronal cell death involve JNK/ERK1/2 signalling and improved hippocampal histone acetylation in neonatal rats. J Pharm Pharmacol 69(6):684–697. 10.1111/jphp.12704 28294340 10.1111/jphp.12704

[CR84] Yang J, Wise L, Fukuchi K-I (2020) TLR4 cross-talk with NLRP3 inflammasome and complement signaling pathways in Alzheimer’s disease. Front Immunol 11:724. 10.3389/fimmu.2020.00724 32391019 10.3389/fimmu.2020.00724 PMC7190872

[CR85] Yang X, Chu S-F, Wang Z-Z, Li F-F, Yuan Y-H, Chen N-H (2021) Ginsenoside Rg1 exerts neuroprotective effects in 3-nitropronpionic acid-induced mouse model of Huntington’s disease via suppressing MAPKs and NF-κB pathways in the striatum. Acta Pharmacol Sin 42(9):1409–1421. 10.1038/s41401-020-00558-4 33214696 10.1038/s41401-020-00558-4 PMC8379213

[CR86] Zhang S, Tang M-B, Luo H-Y, Shi C-H, Xu Y-M (2017) Necroptosis in neurodegenerative diseases: a potential therapeutic target. Cell Death Dis 8(6):e2905–e2905. 10.1038/cddis.2017.286 28661482 10.1038/cddis.2017.286 PMC5520937

[CR87] Zhao X, Fang J, Li S, Gaur U, Xing X, Wang H, Zheng W (2019) Artemisinin attenuated hydrogen peroxide (H(2)O(2))-induced oxidative injury in SH-SY5Y and hippocampal neurons via the activation of AMPK pathway. Int J Mol Sci. 10.3390/ijms20112680 31151322 10.3390/ijms20112680 PMC6600327

[CR88] Zhao X, Huang X, Yang C, Jiang Y, Zhou W, Zheng W (2022) Artemisinin attenuates amyloid-induced brain inflammation and memory impairments by modulating TLR4/NF-κB signaling. Int J Mol Sci. 10.3390/ijms23116354 35683033 10.3390/ijms23116354 PMC9181281

[CR89] Zhou Y, Jin H, Wu Y, Chen L, Bao X, Lu C (2019) Gallic acid protects against ethanol-induced hepatocyte necroptosis via an NRF2-dependent mechanism. Toxicol Vitro 57:226–232. 10.1016/j.tiv.2019.03.008 10.1016/j.tiv.2019.03.008 30853489

