# Supplementary figures and images for: Artemisinin attenuates 3-nitropropionic acid-induced neurodegeneration via HMGB1/TLR4/NF-κB modulation in a rat model of huntington’s disease
Source: Arch Pharm Res. 2026 Mar 22;49(3):375–92. doi: 10.1007/s12272-026-01604-1 (PMC13076369; doi:10.1007/s12272-026-01604-1)

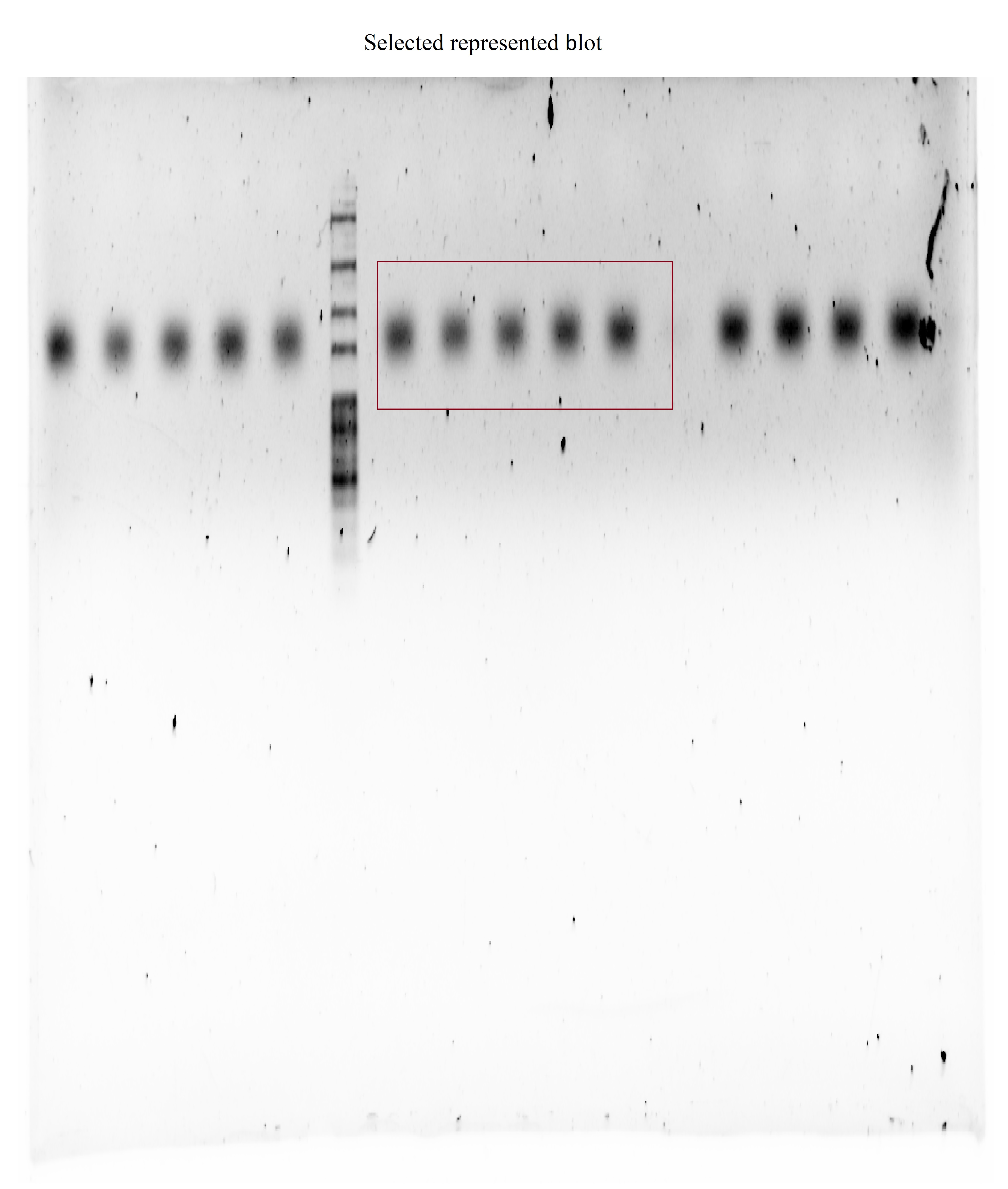

Supplement: Supplementary file 1 — Supplementary file1 (TIF 5769 KB) [file 12272_2026_1604_MOESM1_ESM.tif]

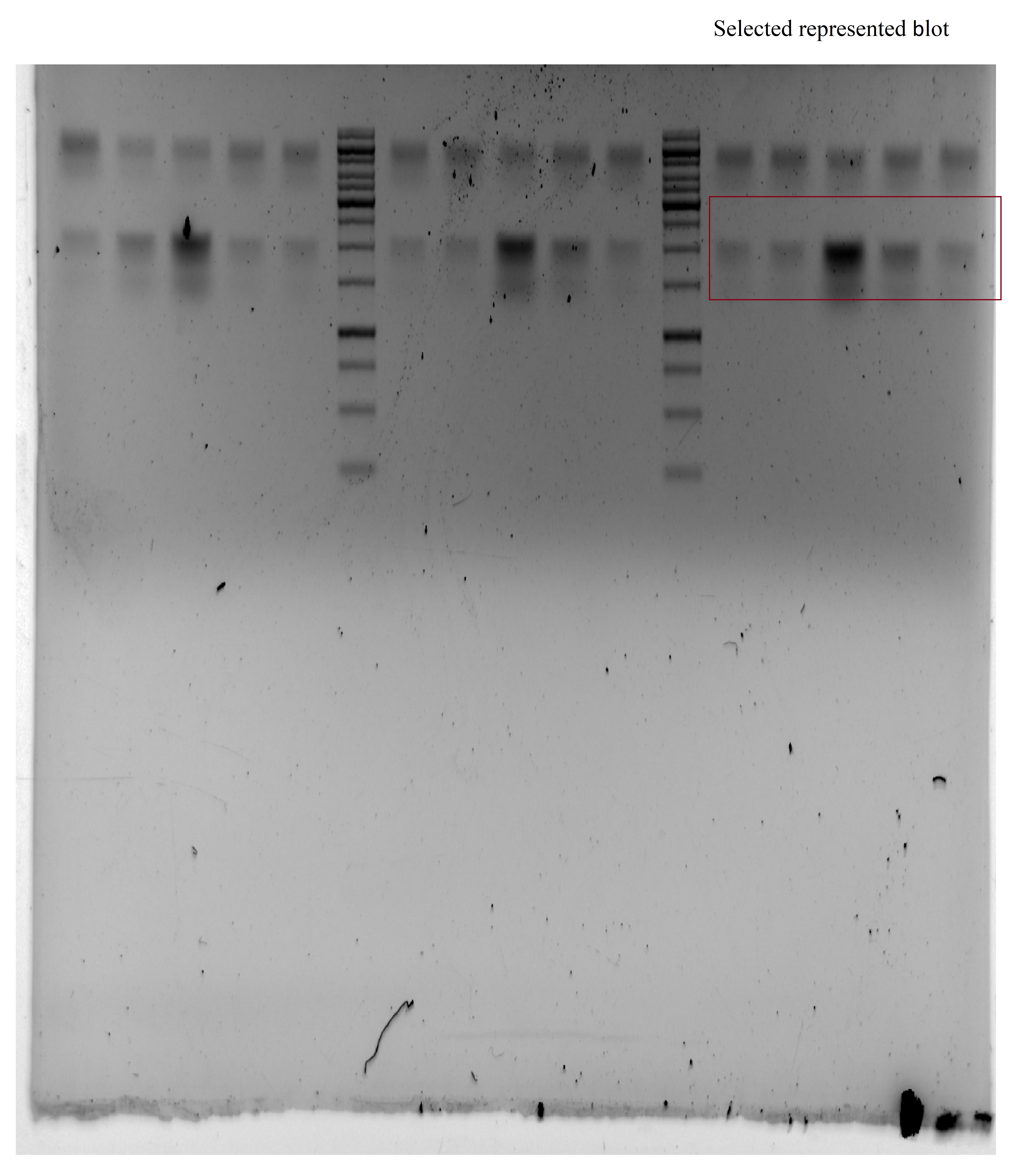

Supplement: Supplementary file 2 — Supplementary file2 (TIF 9336 KB) [file 12272_2026_1604_MOESM2_ESM.tif]

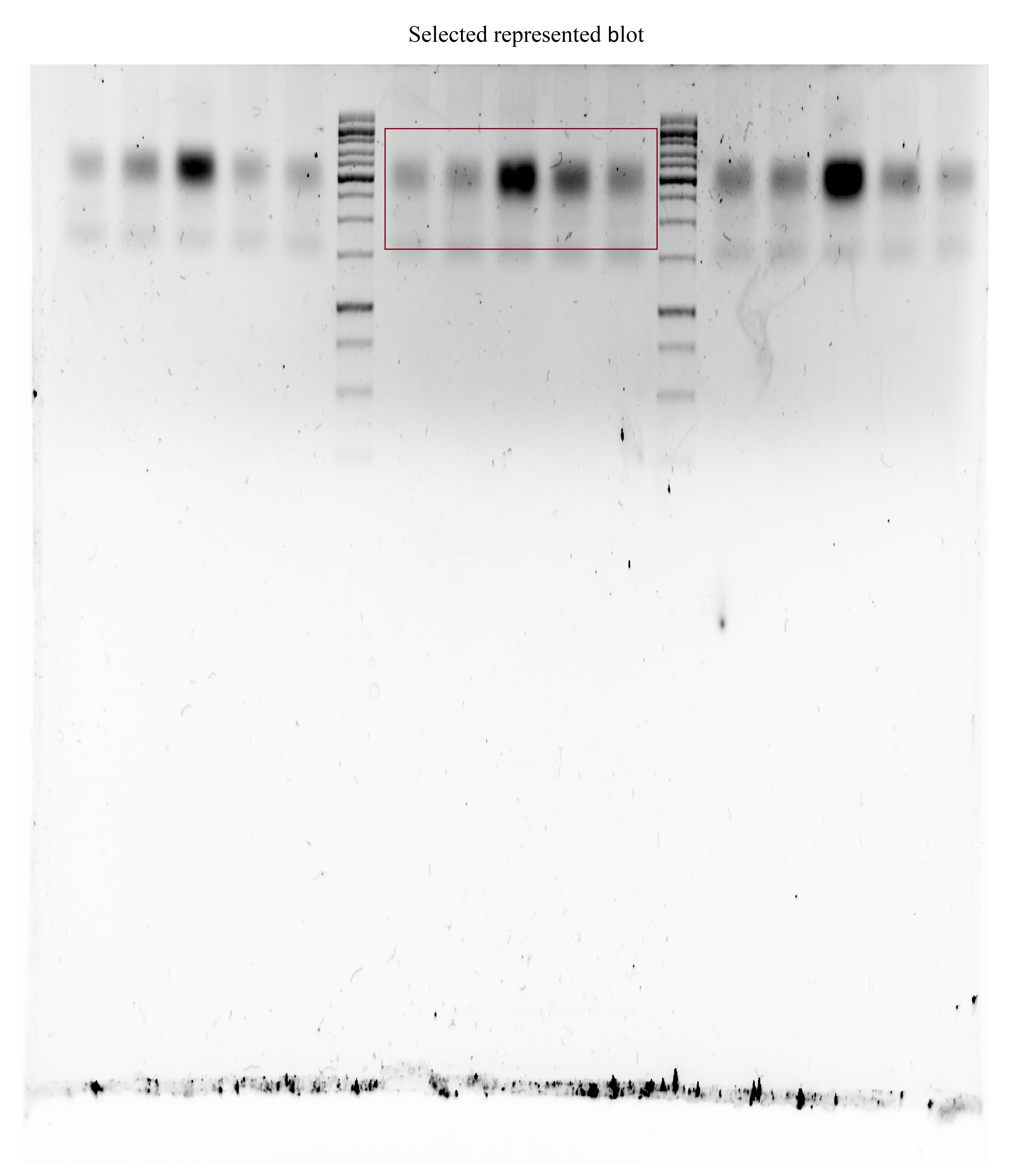

Supplement: Supplementary file 3 — Supplementary file3 (TIF 5917 KB) [file 12272_2026_1604_MOESM3_ESM.tif]

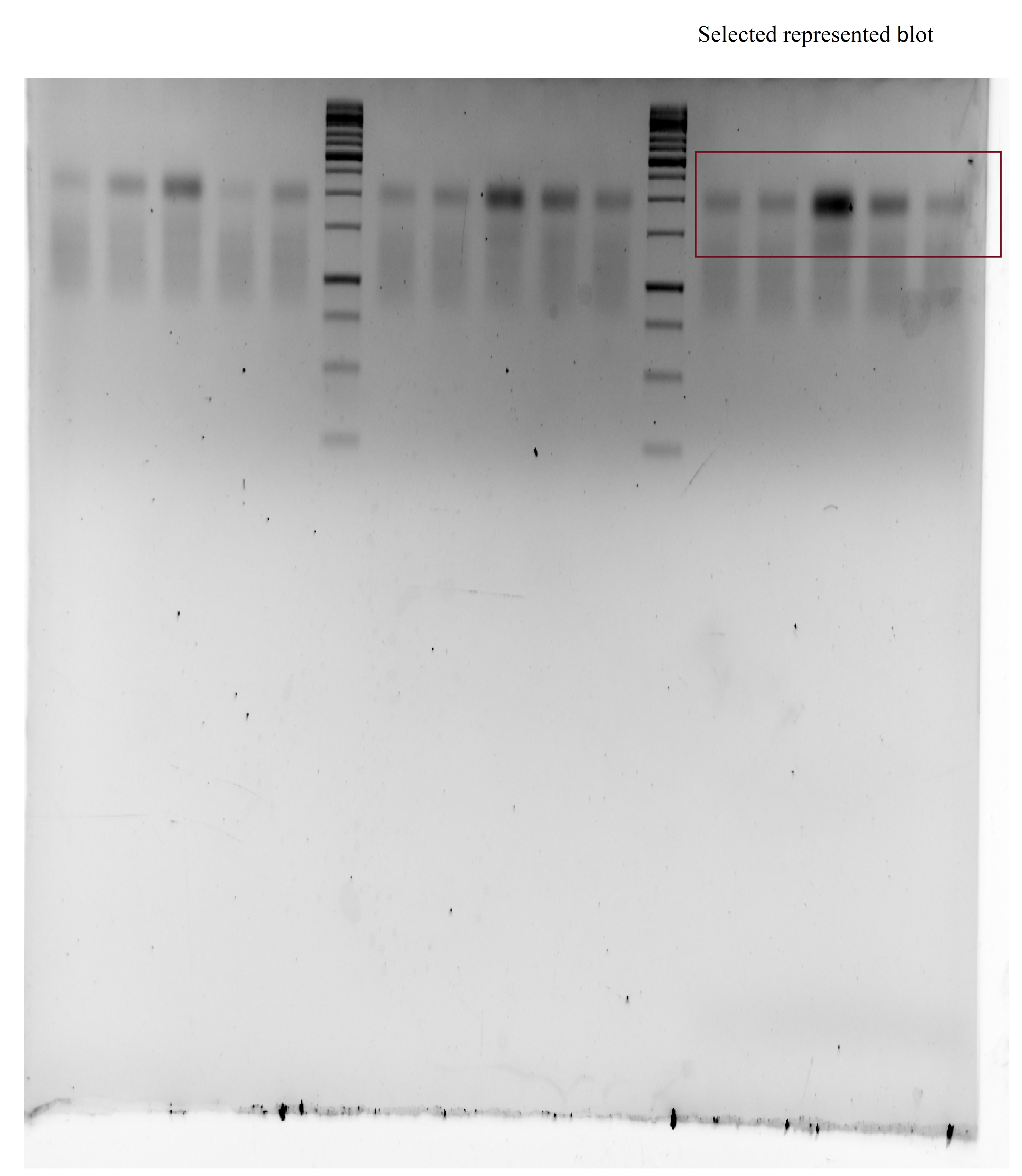

Supplement: Supplementary file 4 — Supplementary file4 (TIF 7901 KB) [file 12272_2026_1604_MOESM4_ESM.tif]

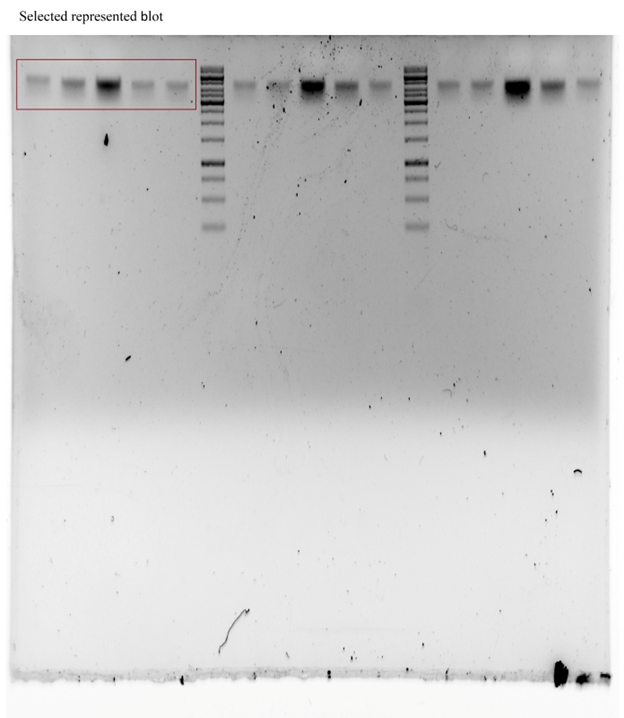

Supplement: Supplementary file 5 — Supplementary file5 (TIF 336 KB) [file 12272_2026_1604_MOESM5_ESM.tif]
