# Supplementary material for: Artemisinin attenuates 3-nitropropionic acid-induced neurodegeneration via HMGB1/TLR4/NF-κB modulation in a rat model of huntington’s disease
Source: Arch Pharm Res. 2026 Mar 22;49(3):375–92. doi: 10.1007/s12272-026-01604-1 (PMC13076369; doi:10.1007/s12272-026-01604-1)
Supplement: Supplementary file 6 — Supplementary file6 (PDF 527 KB) [file 12272_2026_1604_MOESM6_ESM.pdf]

For checking

by Yara Laboud

General metrics

44,683	6,424	308	25 min 41 sec	49 min 24 sec
characters	words	sentences	reading time	speaking time

Score

98

This text scores better than 98% of all texts checked by Grammarly

Writing Issues

26	✓	26
Issues left	Critical	Advanced

Plagiarism

This text hasn't been checked for plagiarism

Writing Issues

3	**Correctness**	
2	Text inconsistencies	
1	Citation style options	
18	**Clarity**	
17	Paragraph can be improved	
1	Intricate text	
4	**Engagement**	
4	Word choice	
1	**Delivery**	
1	Incomplete sentences	

Unique Words

Measures vocabulary diversity by calculating the percentage of words used only once in your document

22%

unique words

Rare Words

Measures depth of vocabulary by identifying words that are not among the 5,000 most common English words.

51%

rare words

Word Length

5.1

Measures average word length

characters per word

Sentence Length

20.9

Measures average sentence length

words per sentence

For checking

Abstract

Huntington's disease (HD) is a progressive neurodegenerative disorder characterized by motor, cognitive, and behavioral impairments associated with striatal neuronal loss, for which effective symptom-attenuating therapies remain lacking. Artemisinin[1] (ART), a natural sesquiterpene lactone with established antioxidant and anti-inflammatory actions, has recently gained attention as a potential neuroprotective agent. This[1] study evaluated the therapeutic relevance of ART in a rat model of HD induced by 3-nitropropionic acid (3-NP). 3-NP[1] administration caused severe behavioral deficits, including an 81.8% reduction in rearing and a 74.9% reduction in ambulation ($p < 0.0001$), a 63.7% decrease in novel object exploration, and a 53.5% decline in Morris water maze target quadrant time versus controls. Biochemically[1], 3-NP elevated HMGB1 (4.8-fold), TLR4 (6.8-fold), RIPK1 (6.4-fold), RIPK3 (5.2-fold), MLKL (5.5-fold), p38-MAPK (4.2-fold), NF-κB (2.1-fold), and TNF-α (4.5-fold), while reducing GSH (57.6%), Nrf2 (77.7%), Sig1R (86.2%), D2R (64%), XIAP (77.8%), BDNF (57.6%) and SDH (61.44%) (all $p < 0.0001$). Treatment[1] with ART (100 mg/kg) markedly restored behavioral performance, increasing rearing and ambulation by 3.2- and 2.6-fold, novel object exploration by 2.4-fold, and target quadrant time by 1.7-fold compared to the 3-NP group. At[1] the molecular level, ART reduced HMGB1 (69.2%), TLR4 (60.4%), RIPK1 (66.3%), RIPK3 (66.4%), MLKL (58%), and TNF-α (62.5%), while significantly restoring GSH (2.1-fold), Nrf2 (3.7-fold), Sig1R (5.2-fold), D2R (2.6-fold), XIAP (3.7-fold), BDNF (2.3-fold) and SDH (1.94-fold) relative to 3-NP-treated rats. Collectively[1], these results demonstrate that ART confers robust neuroprotection against 3-NP-induced

HD-like pathology by attenuating oxidative stress, suppressing HMGB1/TLR4/NF-κB signaling, inhibiting necroptosis, and upregulating neuroprotective markers. These[1] findings highlight ART not only as a neuroprotective agent but also as a promising symptom-attenuating therapeutic candidate for Huntington's disease and other neurodegenerative disorders driven by oxidative and inflammatory stress.

Keywords

Huntington's disease, 3-nitropropionic acid, Artemisinin, neuroinflammation, HMGB1/TLR4/NF-κB, necroptosis.

Introduction

Huntington's disease (HD) is a neurodegenerative disorder marked by progressive loss of striatal neurons, leading to impaired motor function and cognitive decline (Cho, 2012).[2] Clinically,[1] HD presents with uncontrollable movements, such as chorea, along with cognitive deficits and behavioral

disturbances (Kumar et al., 2010).[2] One[1] of the key pathological features of HD is mitochondrial dysfunction, particularly involving impaired energy metabolism (Gao et al., 2015).[2] To[1] investigate the underlying mechanisms of HD-related neurodegeneration, 3-nitropropionic acid (3-NP), a mitochondrial toxin, is commonly used as an experimental model. 3-NP induces striatal damage and motor impairments similar to those observed in HD, making it a valuable tool for studying disease pathophysiology and potential therapeutic interventions (Wu et al., 2010; Ibrahim & Abdel Rasheed, 2022).[2]

Neuroinflammation is another key feature of HD progression. The[1] release of damage-associated molecular patterns (DAMPs), particularly high-mobility group box 1 (HMGB1), activates Toll-like receptor 4 (TLR4), triggering downstream nuclear factor-κB (NF-κB) and mitogen-activated protein kinase (MAPK) pathways (Mustafa et al., 2024).[2] This[1] results in sustained oxidative stress, production of proinflammatory cytokines, and neuronal death. Furthermore,[1] receptor-interacting kinases receptor-interacting protein kinase (RIPK1) and RIPK3 act as central mediators of necroptosis, a regulated form of necrotic cell death that exacerbates striatal injury and fuels inflammatory feedback loops (Wei et al., 2023).[2] The[1] interplay between mitochondrial dysfunction, oxidative stress, and necroptotic signaling provides a strong rationale for targeting these pathways in HD.

Artemisinin (ART), a sesquiterpene lactone derived from Artemisia annua, is well established as a frontline antimalarial agent. However,[1] accumulating evidence highlights its broader pharmacological actions, including potent antioxidant, anti-inflammatory, and antiapoptotic properties (Zhao et al., 2019; Lin et al., 2021).[2] Preclinical[1] studies have demonstrated that ART can modulate TLR4/NF-κB signaling and suppress proinflammatory cytokine release (Zhao et al., 2022).[2] Apart[1] from its anti-inflammatory properties, ART has been

demonstrated to boost the synthesis of antiapoptotic proteins, an especially cellular inhibitor of apoptosis (c-IAP-1, c-IAP-2, and xIAP) (G. Xu et al., 2017), which are known to control RIPK1/RIPK3 levels by promoting their ubiquitination and degradation, so inhibit apoptosis and necroptosis (Lawlor et al., 2015; Ali et al., 2021)[2]. Moreover[1], in models of Alzheimer's and Parkinson's disease, ART treatment has been shown to improve behavioral outcomes and restore neuroprotective markers, supporting its therapeutic relevance beyond infectious disease (Zhao et al., 2019)[2].

Given these findings, we hypothesized that ART could mitigate striatal degeneration and behavioral deficits in HD by targeting oxidative stress, neuroinflammation, and necroptosis pathways. Therefore[1], the present study aimed to evaluate the neuroprotective potential of ART in a 3-NP-induced rat model of HD, with a particular focus on its effects on HMGB1/TLR4/NF-κB signaling and RIPK1/RIPK3-mediated necroptosis.

Materials and Methods

Ethics Statement

The research ethics committee of the faculty of pharmacy at Egyptian Russian University approved all procedures for the care and handling of the animals used in the experiments. Code[1] ERUFP-PO-25-004 was used to approve the study protocol. Efforts[1] were made to alleviate any discomfort the animals may have experienced throughout the study.

Animals

Sixty male albino rats, ranging in weight from 180 to 200 grammes, were used in this research. Located in Cairo, Egypt, El-Nile Company for Pharmaceutical and Chemical Industries supplied the animals. They were given a week to become used to the lab environment before the experiment. The rats were housed in controlled conditions with a 12-hour light-dark cycle, humidity maintained at 60 ± 10%, and a constant temperature of 23 ± 2 °C. A basic chow meal and unlimited water were provided to them.

Experimental Design

Rats were randomly divided into five groups (n = 12 per group). Group I (normal control) received oral saline. Group II was administered ART (100 mg/kg/day, p.o.; Sigma-Aldrich, MO, USA) (Guang Xu et al., 2017), Groups III-V received 3-nitropropionic acid (3-NP; 10 mg/kg/day, i.p.; Sigma-Aldrich, MO, USA) (Mustafa et al., 2021). In addition, Group IV received ART (50 mg/kg/day, p.o.) and Group V received ART (100 mg/kg/day, p.o.) (Guang Xu et al., 2017). Treatments were administered for 14 consecutive days. 3-NP and ART were prepared in saline and adjusted to pH 7.4 with sodium hydroxide.

The animals were tested for behavioral issues on days 15 and 16. Following the evaluations, the animals were put down, their striatum was removed, rinsed with ice-cold saline, flash-frozen in liquid nitrogen, and then stored at -80°C for future analysis. After that, three groups of brains were randomly assigned. After being immersed in RIPA buffer supplemented with protease and phosphatase inhibitors, the first fraction (n = 3/group) was used for western blot analysis. The second group, consisting of six individuals, was examined by dividing the brain into its two halves. For parameter assessment using enzyme-linked immunosorbent assay (ELISA), the striatum in the first hemisphere (n = 6) was homogenised in phosphate-buffered saline (PBS). At the same time, n = 6 of the contralateral hemisphere's striatum were submerged in lysis buffer to evaluate

parameters by RT-qPCR tests. Histological[1] examination of the striatum and immunohistochemical research of Glial Fibrillary Acidic Protein (GFAP) were conducted on the third subgroup (n = 3/group) after preservation in 10% formalin saline. Scheme[1,6] 1.

Scheme 1: Sequence for experimental design, behavioral assessment, and histopathological alteration.

Behavioral Tests

The rats were evaluated for motor function using the open field and rotarod tests 24 hours after the last injection of 3-NP and Artemisinin. The[1] Morris water maze and the Novel Object recognition tests were also used to evaluate memory. The[1] tests were carried out over two days with a 2-hour break in[7] between each day[7] during the light cycle. On[1] the first day, the Morris Water Maze was administered, and on the second day, the Open Field and Novel Object Recognition tests were run (Sayed et al., 2020)[2].

Open Field Test

The open-field test was used to assess spontaneous locomotor activity. Red[1] walls and a black polished floor formed the device's square, with white lines dividing the box into 16 equal squares. This[1] object measured 80 × 80 × 40 cm. The[1] rats were scattered around the field and given five minutes to explore independently. We[1] used the camera to monitor two behavioral variables: the number of squares traversed by[8] each rat (ambulation frequency) and the rate at which each rat stood up[8] on its hind legs (rearing frequency). After[1] examining each animal, the floor was disinfected extensively between trials (Ramachandran & Thangarajan, 2018)[2].

Novel Object Recognition Test

Tests for recognition memory were administered using the Novel Object Recognition (NOR) battery. The[1] experiment was carried out in a black open-field box measuring 50 × 25 × 50 cm. Over[1] two days, rats were habituated by exploring the empty arena for ten minutes each day. The[1] training phase involved placing each rat in an arena with two identical objects spaced approximately 30 cm apart. The[1] day of the test saw the rats returned to the arena, along with the substitution of a new item for an old one. A[1] camera above videotaped the 3-minute exam and training sessions while participants explored each item (Chen et al., 2019).[2] The[1] discrimination index (DI) is calculated as the difference in exploration time between the novel and familiar objects divided by the total exploration time. The[1] total time spent exploring each object was recorded (Mustafa et al., 2021).[2]

Morris Water Maze

The Morris Water Maze (MWM) was used to evaluate spatial learning and memory. The[1] experiment was carried out in a circular pool with non-reflective inner surfaces, measuring 150 cm in diameter and 60 cm in height. The[1] pool was filled to a depth of 35 cm with water kept at 25 ± 2 °C. A[1] 9-centimeter-diameter escape platform was placed one centimeter below the water's surface in the middle of one of the four pool corners identified as the target area. We[1] applied non-toxic black paint to the water to make the platform invisible. Rats[1] underwent acquisition training for 4 days in a row,[9] with 3 trials per day (120 seconds per trial), beginning at different locations. If[1] the platform could not be found during the trial, the rat was led to it and left for 30 seconds. The[1] average time it took to reach the platform across trials was called acquisition latency. The[1] platform was removed on day five to conduct a probing trial. After[1] positioning themselves in the quadrant across from the goal, the rats were given 60 seconds to swim. To[1] measure memorization, a camera

mounted above the head tracked how much time was spent in each quadrant (Suganya & Sumathi, 2017)[2]

Biochemical Parameters

Western blot analysis of TLR4, RIPK1, RIPK3 and p38 (pT180/Y182)-MAPK

The Striatal tissues were ground in RIPA buffer, and the Bradford Protein Assay Kit (Bio BASIC Inc.[10], ON, Canada) was used to measure total protein content. Protein[1] concentrations of 10 µg for each[11] sample were deposited onto[11] PVDF membranes after SDS-PAGE separation and were[11] blocked with 5% BSA. Thermo[1] Fisher Scientific, MA, USA, provided the primary antibodies to incubate the membranes. These antibodies were specific for TLR4 TLR4 (2 µg/ml; cat#: PA5-23124), phospho-p38 MAPK (Thr180/Tyr182), (1:1000; cat#: 44-684G), RIPK1 (1 µg/mL; cat# PA5-20811), RIPK3 (0.1-0.5 µg/mL; cat# PA5-19956), and β-actin (1:1000; cat# PA5-16914).[1] Afterward, membranes were probed with horseradish peroxidase-conjugated goat anti-rabbit immunoglobulin (Dianova, Hamburg, Germany) for 2 h at room temperature. All[1] antibodies were validated by the supplier for specificity, and protein bands were detected at their expected molecular weights. The[1] expression of the target protein was measured by densitometric analysis in Image Lab software on the ChemiDoc™ MP Imaging System (version 3) (Bio-Rad, CA, United States). The[1] percentage of acrylamide used for all studied proteins was 10%. The[1] outcomes were normalized to β-actin and presented in arbitrary units (AU).

ELISA assay

The following enzyme-linked immunosorbent assays (ELISA) were used to quantify BDNF, GSH, HMGB1, NF-κB p65, MDA, 8-OHdG, and SDH in phosphate-buffered saline (PBS): (cat# MBS2019439), (cat# MBS9712516), (cat# MBS703437), (cat# MBS2505513), (cat# MBS738685), (cat# MBS267513), and (cat# MBS3807968) from MyBioSource, Irvine, USA. A[1] Cusabio ELISA kit (cat#

CSB-E11987r) from Wuhan, PRC was used to test TNF-α. All[1] tests were carried out according to the manufacturer's instructions. Results[1] for BDNF, NF-κB p65, TNF-α, and 8-OHdG were expressed as pg/mg of tissue protein, whereas GSH, HMGB1, MDA, and SDH levels were reported as ng/mg of tissue protein.

Quantitative RT-PCR

Brain tissues were homogenized for mRNA extraction. Total[1] RNA was isolated, and cDNA was synthesized using a reverse transcription system. Quantitative[1] RT-PCR was conducted for XIAP, Nrf2, D2R, Sig1R, and MLKL using SYBR Green Master Mix. Amplification[1] was performed for 40 thermal cycles under optimized conditions. Primer[1] specificity was confirmed by melt-curve analysis, which demonstrated a single sharp peak for each target, indicating the absence of non-specific amplification or primer-dimer formation.

Amplification efficiencies for all primer pairs were validated using standard curve analysis and were within the acceptable range (90-110%), allowing the use of the comparative cycle threshold (2^-ΔΔCt) method. The[1] housekeeping gene GAPDH was selected as an internal reference, and its expression was verified to be stable across all experimental groups. Relative[1] gene expression levels were calculated using the Ct method, normalized to GAPDH, and expressed as fold changes relative to the control group.

Histopathological examination.

Over 72 hours, with solution changes made daily, tissue samples were fixed in 10% neutral buffered formalin. Rinsing[1], dehydration using a graded ethanol series, xylene clearing, infiltration with synthetic wax, and embedding in Paraplast tissue embedding medium, followed by fixation. Light[1] microscopy was used to analyze sagittal brain slices (5 μm) of the striatum that had been cut using a rotary microtome and stained with Hematoxylin and Eosin (H&E) (Sidhu et al., 2018)[2]. Using[1] the Leica application module for histological analysis,

all micrographs and data were collected with a full-HD microscope camera (Leica Microsystems GmbH, Wetzlar, Germany).

Immunohistochemical detection of GFAP

Paraffin-embedded sections were mounted on positively charged slides and processed using the avidin-biotin-peroxidase complex (ABC) method. Following incubation with primary monoclonal antibodies, sections were treated with reagents from the Vectastain ABC-HRP kit (Vector Labs). Antigen-antibody binding was visualized by peroxidase-mediated diaminobenzidine (DAB; Sigma) staining. Non-immune serum replaced primary and secondary antibodies in negative control slides. Immunostained sections were examined using an Olympus BX-53 microscope. Immunoreactivity was quantified by analyzing the percentage area of positive staining in 10 randomly selected fields per section using ImageJ (version 1.53t), as described by Rasband et al. (NIH, USA).

Statistical Analysis

Data are presented as mean ± standard deviation. A one-way ANOVA followed by Tukey's multiple-comparison test was applied to most parameters. Ambulation and rearing frequency were analyzed using the Kruskal-Wallis test with Dunn's post hoc comparisons. A two-way ANOVA was performed within groups to assess differences in exploration time between familiar and novel objects, accounting for both object type and treatment effects. Statistical analyses were conducted using GraphPad Prism version 9.

Results

Effect of Artemisinin on Behavioural and Motor Alterations in the 3-NP rat model.

The Novel Object Recognition test assessed spatial and non-spatial memory in rats. Statistical[1] analysis showed significant differences in both the discrimination index (F(4, 55) = 87.44, $p < 0.0001$) and total exploration time for novel versus familiar objects (F(4, 55) = 13.98, $p < 0.0001$).

Administration of 3-NP resulted in a significant decline in the discrimination index (-0.18, $P < 0.0001$) and a (41.55%, $P < 0.0001$) reduction in total exploration time compared to the control group. However[1], cotreatment with 50 mg and 100 mg ART significantly improved the discrimination index (0.22, $P < 0.0001$) and (0.26, $P < 0.0001$), respectively, relative to the 3-NP group. Additionally[1], co-administration of 50 mg and 100 mg ART significantly increased the total time spent exploring both objects (1.5-fold, $P = 0.0001$) and (1.54-fold, $P < 0.0001$), respectively, compared to the diseased group.

Further analysis revealed that the 3-NP group showed a significant 63.65% reduction ($p < 0.0001$) in time spent exploring the novel object compared to controls. Treatment[1] with ART at 50 mg and 100 mg doses significantly increased novel object exploration by 2.27-fold and 2.36-fold, respectively ($p < 0.0001$ vs. 3-NP group). No[1] significant differences were found among groups in the time spent exploring the familiar object.

The open field test assessed locomotor and hyperactivity behaviors by measuring rearing and ambulation frequencies. Administration[1] of 3-NP significantly reduced rearing and ambulation by 81.79% and 74.94%, respectively ($p < 0.0001$ vs. control). Treatment[1] with 100 mg ART significantly restored rearing and ambulation frequencies by 3.17-fold ($p = 0.0152$) and 2.59-fold ($p = 0.0073$), respectively, compared to the 3-NP group.

The Morris Water Maze was employed to evaluate spatial memory and locomotor function. Statistical analysis revealed significant differences in time spent in the target quadrant across groups (F (4, 55) = 35.56, p < 0.0001). 3-NP administration significantly decreased target quadrant time by 53.47% (p < 0.0001) relative to controls. Treatment with ART at 50 mg and 100 mg doses significantly increased target quadrant time by 1.53-fold (p = 0.0001) and 1.67-fold (p < 0.0001), respectively, compared to the 3-NP group. Figure. 1

Effect of Artemisinin on 3-NP-induced Striatal Histopathological Alterations.

Histopathological examination of the striatum in all groups was performed using H&E staining. The striatum exhibited a normal histological structure in both the control and the ART-only treated group. However, the administration of 3-NP resulted in capillary congestion accompanied by hemorrhage, severe perineuronal edema, and pronounced astrogliosis. Interestingly, in the group treated with 50 mg of ART, the striatum showed moderate perineuronal edema with severe astrogliosis. Similarly, cotreatment with 100 mg of ART further improved the condition, resulting in mild perineuronal edema with severe astrogliosis. Figure. 2

Effect of Artemisinin on 3-NP-induced changes in striatal GFAP immunoreactivity.

The immunoreactivity of striatal GFAP was evaluated through immunostaining to assess the extent of astrocyte activation. Statistical analysis using one-way ANOVA revealed a significant difference between the groups (GFAP: F (4, 45) = 97.07, P < 0.0001). In the control group and the ART-only group, no detectable GFAP expression was observed in the striatum. However, exposure to 3-NP resulted in a significant increase in GFAP levels (P < 0.0001, 18.24-fold) compared with the control group, indicating strong astrocyte activation. Interestingly, treatment with a low dose of ART (50 mg) significantly reduced

GFAP expression by (P < 0.0001, 30.13) compared to the 3-NP group. This reduction was even more pronounced with a higher dose of ART (100 mg), which significantly decreased GFAP expression by 56.05% (P < 0.0001) relative to the 3-NP group. Notably, the 100 mg ART dose also showed a significant (P = 0.0006, 37.1%) reduction in GFAP levels compared with the 50 mg ART dose group, highlighting the dose-dependent effect of ART in suppressing astrocyte activation. Figure. 3

Effect of Artemisinin on striatal contents of TLR4, RIPK1, RIPK3, MLKL, p38 (pT180/Y182)-MAPK, HMGB1 in 3-NP rat model.

In current study, statistical analysis revealed significant differences among the groups in the levels of TLR4, RIPK1, RIPK3, MLKL, p38 (pT180/Y182)-MAPK, and HMGB1 (TLR4: F (4, 10) = 21.7, P < 0.0001), (RIPK1: F (4, 10) = 205.5, P < 0.0001), (RIPK3: F (4, 10) = 18.11, P = 0.0001), (MLKL: F (4, 10) = 59.84, P = 0.0001), (p38 (pT180/Y182)-MAPK: F (4, 10) = 41.7, P < 0.0001) and (HMGB1: F (4, 10) = 169.8, P < 0.0001), intoxication with 3-NP significantly elevated the levels of TLR4 (P < 0.0001, 6.84-fold), RIPK1 (P < 0.0001, 6.36-fold), RIPK3 (P = 0.0002, 5.2-fold), MLKL (P = 0.0001, 5.5-fold), p38MAPK (P < 0.0001, 4.22-fold), and HMGB1 (P < 0.0001, 4.83-fold) compared to the control group.

However, treatment with 50 mg of ART significantly reduced the levels of TLR4 (P = 0.0029, 55.16%), RIPK1 (P < 0.0001, 50.51%), RIPK3 (P = 0.0053, 53.06%), MLKL (P = 0.0001, 41.6%), p38 (pT180/Y182)-MAPK (P = 0.0002, 49.22%), and HMGB1 (P < 0.0001, 51.52%) relative to the 3-NP intoxicated group. Similarly, co-administration of 100 mg of ART resulted in a significant decrease in the levels of TLR4 (P = 0.0015, 60.39%), RIPK1 (P < 0.0001, 66.34%), RIPK3 (P = 0.001, 66.41%), MLKL (P = 0.0001, 58%), p38 (pT180/Y182)-MAPK (P < 0.0001, 61.7%), and HMGB1 (P < 0.0001, 69.17%) compared to the 3-NP group. The higher dose of 100 mg ART significantly reduced RIPK1 levels by 31.97% (P =

0.0068) and HMGB1 levels by 36.42% ($P = 0.0004$) compared to the 50 mg ART-treated group. Figure.[1] 4

Effect of Artemisinin on striatal GSH, Nrf2, MDA, and 8-OHDG in 3-NP rat model.

Striatal levels of antioxidants GSH, Nrf2, MDA, and 8-OHdG were analyzed. One-way[1] ANOVA demonstrated significant differences among groups for all markers: GSH ($F(4, 25) = 35.65$, $p < 0.0001$), Nrf2 ($F(4, 25) = 222.3$, $p < 0.0001$), MDA ($F(4, 25) = 153.3$, $p < 0.0001$), and 8-OHdG ($F(4, 25) = 287.3$, $p < 0.0001$). 3-NP administration significantly decreased GSH and Nrf2 levels by 57.6% and 77.72%, respectively ($p < 0.0001$ vs. control), while markedly increasing MDA and 8-OHdG levels by 4.33 and 4.81-fold ($p < 0.0001$). Treatment[1] with 50 mg ART significantly restored GSH and Nrf2 levels by 1.91-fold and 3.17-fold, respectively ($p < 0.0001$ vs. 3-NP), and reduced MDA and 8-OHdG levels by 45.9% and 64.5% compared to the 3-NP group ($p < 0.0001$).

Co-administration of 100 mg ART significantly elevated GSH and Nrf2 levels by 2.13-fold and 3.73-fold, respectively ($p < 0.0001$ vs. 3-NP), and reduced MDA and 8-OHdG by 61.5% and 72.51% ($p < 0.0001$). Furthermore,[1] the 100 mg dose increased Nrf2 expression by 1.17-fold compared to the 50 mg ART-treated group ($p < 0.0001$). Figure.[1] 5

Effect of Artemisinin on striatal neuroinflammatory markers and SDH in 3-NP rat model.

Striatal NF-Kb, TNF-α, and SDH were quantified. One-way[1] ANOVA revealed significant differences among groups for NF-κB ($F(4, 25) = 187.3$, $p < 0.0001$), TNF-α ($F(4, 25) = 174.7$, $p < 0.0001$), and SDH ($F(4, 25) = 67.42$, $p < 0.0001$). 3-NP intoxication significantly elevated NF-Kb and TNF-α levels by 2.09-fold and 4.52-fold, respectively, while decreasing SDH level by 61.44% ($p < 0.0001$ vs. control). Treatment[1] with 50 mg ART significantly decreased NF-κB by

36.25% and TNF-α by 45.53% and increased SDH to 1.48-fold compared to the 3-NP group ($p < 0.0001$).

Co-administration of 100 mg ART significantly decreased NF-κB and TNF-α levels by 44.02% and 62.49%, respectively, and increased SDH level to 1.94-fold ($p < 0.0001$ vs. 3-NP). The 100 mg dose also reduced TNF-α by 31.12% compared to the 50 mg ART group ($p = 0.005$). Figure. 6

Effect of Artemisinin on striatal Sig1, D2R, XIAP, and BDNF in 3-NP rat model.

Sig1, D2 receptor, XIAP, and BDNF are neuroprotective in various neurodegenerative diseases. In this study, statistical analysis identified significant differences among the experimental groups in the expression levels of these proteins: (Sig1: $F(4, 25) = 251.1$, $P < 0.0001$), (D2 receptor: $F(4, 25) = 222.2$, $P < 0.0001$), (XIAP: $F(4, 25) = 222.3$, $P < 0.0001$), and (BDNF: $F(4, 25) = 52.98$, $P < 0.0001$).

3-NP exposure significantly decreased Sig1 (86.21%), D2 receptor (64%), XIAP (77.75%), and BDNF (57.61%) levels compared to controls ($p < 0.0001$). Treatment with 50 mg ART significantly restored these markers by 4.57-fold (Sig1), 2.13-fold (D2 receptor), 3.15-fold (XIAP), and 2.18-fold (BDNF) relative to the 3-NP group ($p < 0.0001$).

Treatment with 100 mg ART significantly upregulated Sig1 (5.15-fold), D2 receptor (2.56-fold), XIAP (3.71-fold), and BDNF (2.29-fold) compared to the 3-NP group ($p < 0.0001$). Additionally, the 100 mg dose further increased D2 receptor (1.19-fold, $p < 0.0001$) and XIAP (1.17-fold, $p = 0.003$) expression relative to the 50 mg ART-treated group. Figure. 7

Discussion

This work presents initial evidence of the neuroprotective effect of ART against 3-NP-induced neurotoxicity in a rat model. Several lines of evidence

substantiated the protective effect: (i) enhancement of motor and cognitive performance, (ii) reduction of oxidative stress, indicated by elevated levels of Nrf2, GSH, and decrease 8-OHdG, (iii) suppression of neuroinflammatory markers, such as NF-κB p65 and TNF-α, (iv) downregulation of HMGB1/TLR4 signaling, correlated with diminished expression of the downstream RIPK1/RIPK3/p38-MAPK axis, and (v) upregulation of the antiapoptotic protein XIAP and mitochondrial complex II, succinate dehydrogenase (SDH).

The striatum, a key component of the basal ganglia, is essential for motor coordination, learning, and memory (Jang & Cho, 2016).[2] 3-NP,[1] a mitochondrial toxin, has been widely used to create experimental models that mimic the clinical and pathological characteristics of Huntington's disease. The[1] administration of 3-NP induces selective neurodegeneration, predominantly targeting medium spiny neurons in the striatum, resulting in motor dysfunction, cognitive impairments, and compromised memory retention (Kumar et al., 2007b; Aziz et al., 2008).[2] 3-NP[1] generated hippocampal lesions in the CA1 and CA3 pyramidal neuron areas, closely linked to cognitive function (Kumar & Kumar, 2009),[2] and elevated acetylcholinesterase activity, exacerbating cognitive loss. Besides[1] the striatum, 3-NP has been shown to impair several brain areas, including the hippocampus, thalamus, and cerebral cortex (Borlongan et al., 1997).[2] Behavioral[1] research has revealed that mice treated with 3-NP exhibit marked reductions in locomotor activity, compromised coordination, and altered limb reflexes (Kumar et al., 2007a; Bhateja et al., 2012).[2]

Furthermore, histopathological analysis indicates that 3-NP exposure results in significant neuronal degeneration within the lesion core and heterogeneous neuronal reactions in the adjacent transition zone, characterized by pronounced damage to projection neurons. In[1] contrast, interneurons may

exhibit proliferative alterations (Mu et al., 2011; Tasset et al., 2011).[2] The[1] neurotoxicity induced by 3-NP is primarily attributed to mitochondrial dysfunction, oxidative stress, disruption of energy metabolism, and the activation[17] of apoptotic pathways, all of which led to the behavioral and structural deficits seen in human and animal models of Huntington's disease (Rosenstock et al., 2004; Sandhir & Mehrotra, 2013).[2]

This study employed behavioral assays, including the open field, Morris water maze, and novel object recognition tests, to comprehensively evaluate 3-NP-induced motor and cognitive impairments in rats, modeling neurodegenerative deficits characteristic of Huntington's disease. ART[1] treatment significantly mitigated these motor and cognitive[18] dysfunctions, demonstrating its neuroprotective potential.

Notably, elevated levels of oxidative stress markers, such as malondialdehyde (MDA) and 8-hydroxydeoxyguanosine (8-OHdG), are consistently observed in neurodegenerative brain regions[19] of patients with Huntington's disease (Klepac et al., 2007).[2] Postmortem[1] investigations provide robust evidence of its role in HD development. Significantly[1] elevated levels of 8-OHdG have been detected in the striatum and forebrain of R6/2 HD mice and peripheral fluids, including urine and plasma, signifying extensive oxidative DNA damage (Chen, 2011).[2] Current[1] data indicate that oxidative stress is intricately associated with disease development and may function as a significant biomarker and therapeutic target in Huntington's disease.

Moreover, Nuclear factor erythroid two-related factor 2 (Nrf2) is a principal regulator of cellular protection against oxidative stress, inflammation, and mitochondrial dysfunction, which are critical characteristics of neurodegenerative disorders, including HD. Nrf2[1] governs the expression of various genes implicated in antioxidant responses, detoxification, the

clearance of damaged proteins and organelles, and energy metabolism (Luchkova et al., 2024).[2] Its[1] malfunction has been associated with HD and Alzheimer's disease. Preclinical[1] studies indicate that pharmacological stimulation of Nrf2 improves cellular resilience, postpones disease onset, and reduces neuronal loss. Pre-symptomatic[1] activation[17] of the Nrf2 pathway may mitigate or avert striatal degeneration in HD, rendering it a persuasive treatment approach for many neurodegenerative disorders (Tucci et al., 2022).[2] Recently,[1] Nrf2 has been identified as a novel regulator of necroptosis, and its pharmacological activation[17] was shown to significantly reduce RIP1 and RIP3 expression in ethanol-treated hepatocytes, thereby inhibiting hepatocyte necroptosis (Zhou et al., 2019).[2]

In line with a previous study, ART treatment significantly ameliorated oxidative stress, as indicated by increased Nrf2 levels and downregulation of[20] 8-OHdG (Deng et al., 2025).[2]

In addition to the changes in GSH, Nrf2, and 8-OHdG, our findings also demonstrated significant alterations in MDA levels, a well-established marker of lipid peroxidation. The[1] elevation of MDA in the 3-NP group confirms the contribution of lipid peroxidation to oxidative damage in Huntington's disease models. Notably,[1] ART treatment markedly reduced MDA levels, highlighting its ability to attenuate lipid peroxidation and[21] further supporting its role as an effective antioxidant and neuroprotective agent.

High-mobility group (HMG) proteins are non-histone DNA-binding proteins ubiquitously expressed in eukaryotic cells and play key roles in diverse biological processes (Dumitriu et al., 2005).[2] Among[1] these, HMGB1 is the most prevalent member, widely distributed across human cells (Ge et al., 2021).[2] In[1] the CNS, HMGB1 is expressed in neurons, microglia, and astrocytes (Manivannan et al., 2021; Huang et al., 2023).[2] When[1] localized on the neuronal

cell surface or within the extracellular matrix, HMGB1 facilitates neurite outgrowth, enhances cell migration, and plays a crucial role in neuroinflammatory responses following injury (Wan et al., 2020). During cellular events such as apoptosis, necrosis, and pyroptosis, HMGB1 is actively translocated into the extracellular space (R. Chen et al., 2022). HMGB1 functions as a DAMP in this extracellular milieu, triggering innate immune Activation by recruiting inflammatory and immune cells. Additionally, it stimulates macrophages and endothelial cells to release proinflammatory cytokines, thereby amplifying the inflammatory response (Ren et al., 2023; Li et al., 2024). Furthermore, once immune and endothelial cells are activated by HMGB1, they can subsequently release HMGB1 themselves, perpetuating a self-sustaining positive feedback loop (Kang et al., 2014) in accordance with the previous study that reported that apoptotic and autophagic pathways are activated in the 3-NP model of HD, which is associated with increased expression of HMGB1. This, in turn, causes damage to neurons (Gendy et al., 2023). The present study demonstrated a significant increase in HMGB1, which ART treatment inhibited.

Beyond its role in neuroinflammation, HMGB1 is a key mediator of inflammatory responses through its interaction with TLR4 (R. Chen et al., 2022). Its involvement has been implicated in various neurological conditions, including epilepsy and neurodegenerative disorders such as HD (Angelopoulou et al., 2020). Moreover, HMGB1 has been shown to upregulate TNF-α, suggesting its role in apoptosis induction via inflammatory pathways (Song et al., 2021). TLR4 is a key player in the body's immune defense system, acting as a pattern recognition receptor (PRR) that detects DAMPs (Kielian, 2006; Venegas & Heneka, 2017). TLR4 triggers a signaling cascade that activates specific transcription factors, leading to the production of cytokines, chemokines,

growth factors, and other inflammatory mediators (Yang et al., 2020)[2]. This[1] response is essential for immune defense but can contribute to chronic inflammation and neurodegenerative diseases when dysregulated.

TLR4 signaling follows two major pathways: the MyD88-TNF receptor-associated factor 6 (TRAF6)-TAK1 pathway and the TRIF-TBK1 pathway. The[1] MyD88-TRAF6 pathway is crucial because it activates TAK1, which regulates p38-MAPK, extracellular signal-regulated kinase (ERK), and NF-κB (Iwamoto et al., 2020)[2].

NF-κB plays a central role in inflammation, acting as a master regulator of immune responses. Under[1] normal conditions, it remains inactive in the cytoplasm, bound to its inhibitor, IκB. However[1], when the immune system detects a threat, IκB is phosphorylated and degraded, freeing NF-κB to enter the nucleus and switch on genes responsible for inflammation (Cui et al., 2010; Napetschnig & Wu, 2013; Rodrigues et al., 2022; D. Chen et al., 2022)[2]. TLR4[1] enhances this process by activating the IκB kinase (IKK) complex, which facilitates NF-κB nuclear translocation and amplifies the inflammatory response (Wang et al., 2019)[2].

In addition to NF-κB activation, TLR4 signaling triggers the MAPK pathway, strengthening the inflammatory response by phosphorylating key proteins (Medvedev et al., 2007; Mitchell et al., 2018)[2]. This[1] activation[17] induces nuclear translocation of transcription factors, including AP-1, c-Fos, c-Jun, ELK-2, and ATF-2, which collectively enhance proinflammatory cytokine expression (Svensson et al., 2011; Patel et al., 2012; Li et al., 2022)[2]. Together[1], NF-κB and p38 MAPK activation drive the release of inflammatory molecules like TNF-α, IL-1β, IL-6, IL-8, and IL-12, a process particularly prominent in microglia, the brain's resident immune cells (Landström, 2010; Feng et al., 2014; Mustafa et al., 2024)[2]. Interestingly[1], 3-NP has previously been reported to activate

TLR4/NF-κB signaling and to increase p38-MAPK expression (Yang et al., 2021; Gendy et al., 2023)[2]. Indeed[1], 3-NP rats showed marked elevation in the signaling mentioned above. Controversy[1]: ART administration inhibited the 3-NP effect. Necroptosis is a distinct type of programmed cell death that displays attributes of both apoptosis and necrosis, usually occurring when apoptotic pathways are impaired (Dhuriya & Sharma, 2018)[2]. In[1] contrast to apoptosis, necroptosis occurs independently of essential apoptotic regulators, including caspases, B-cell lymphoma 2 (Bcl-2), and mitochondrial cytochrome c release (Degterev et al., 2008)[2]. This[1] type of cell death serves as an alternative mechanism, especially in apoptosis-resistant contexts, offering a potential benefit for addressing resistance challenges frequently encountered in cancer treatment (Wu et al., 2020)[2]. Moreover[1], neuronal cell death and neuroinflammation in the pathogenesis of certain neurodegenerative diseases may be facilitated by necroptosis. An[1] inflammatory reaction is triggered by massive amounts[23] of DAMPs released[23] from necroptotic cells (Zhang et al., 2017)[2]. Receptor-interacting protein kinases 1 and 3 (RIPK1 and RIPK3) are crucial in necroptosis (Silke et al., 2015)[2]. Several[1] stimuli trigger them, including DAMPs, TNF, IFN-α, TLRs, viral infections, and genotoxic stresses. TNF-α[1] initiates the most well-defined necroptotic pathway (Thapa et al., 2013)[2], leading to the formation of an amyloid-like complex known as the necrosome, composed of RIPK1 and RIPK3 (Cho et al., 2009)[2]. The[1] Activation and phosphorylation of RIPK1 commence the necroptosis cascade. Activated[1] RIPK1 subsequently activates the downstream target RIPK3, which polymerizes and converges with RIPK1 to form[24] a necrosome complex. RIPK3[1] within the necrosome recruits and phosphorylates its downstream target, including MLKL (Abdel-Magid, 2025)[2]. These[1] kinases also modulate proinflammatory signaling, which is vital for innate immune protection (Kawai & Akira, 2010)[2]. A[1] previous study reported that

3-NP induced striatal toxicity via stimulating the necroptosis pathways involving RIPK1, RIPK3, and MLKL signaling in rats (Elbaz et al., 2025). In the current study, ART demonstrated antiapoptotic and neuroprotective effects by reversing the 3-NP-induced effects and modulating RIPK1 and RIPK3 expression.

Inhibitors of apoptosis (IAP) proteins, including XIAP, IAP1, and IAP2, modulate critical phases of apoptosis by directly inhibiting caspases 3, 7, and 9. These proteins inhibit the amplification of apoptotic signaling mediated by caspase-3. Although IAPs may inhibit apoptosis under various stimuli, their efficacy is diminished in neurodegenerative disorders (Korhonen, 2002). For example, XIAP overexpression protects neurons in ischemia and Parkinson's disease models. In contrast, its degradation has been observed in amyotrophic lateral sclerosis, suggesting that diminished IAP activity may play a significant role in neuronal damage in HD (Goffredo et al., 2005). Herein, 3-NP significantly reduced XIAP expression. At the same time, ART ameliorated the 3-NP effect by elevating XIAP gene expression in striatal tissue.

Brain-derived neurotrophic factor (BDNF), a prominent and well-researched neurotrophin in the mammalian brain, is essential for the functioning of the peripheral and central nervous systems (Mori et al., 2021). Changes in BDNF levels have been suggested as biomarkers for many neurodegenerative diseases. The BDNF/TrkB signaling system is crucial for cerebral development, neuronal plasticity, and functions such as neurogenesis, cellular differentiation, survival, synaptic plasticity, and responses to inflammation, pain, and sensory maturation (Dincheva et al., 2016). Significantly, BDNF has robust neuroprotective and regenerative properties, augmenting neuronal resilience and recovery after damage or degeneration (Pisani et al., 2023). Consistent with previous in vivo studies, in the current investigation, 3-NP rats

demonstrated neurodegeneration, as evidenced by a marked decrease in BDNF levels (Mustafa et al., 2021)[2]. On[1] the other hand, ART showed neuroprotective effects by increasing BDNF levels.

Interestingly, TLR4 activation is also linked to downregulation of the Sigma-1 receptor (Sig1R) via the p38-MAPK and NF-κB pathways. Sig1R[1] is a multifunctional protein that protects brain health (Iwamoto et al., 2020)[2]. Sig1R[1] is mainly found at the junction between the endoplasmic reticulum (ER) and mitochondria, an area known as the mitochondria-associated ER membrane (MAM). It[1] helps regulate cellular stress responses, synaptic communication, ion channel function, and gene expression (Nakamura et al., 2019; Schmidt & Kruse, 2019)[2]. Recent[1] studies suggest that Sig1R agonists can reduce the production of proinflammatory cytokines in microglia, highlighting their potential neuroprotective effects (Wu et al., 2015)[2]. Notably[1], lower levels of Sig1R have been observed in patients with Alzheimer's disease (AD), Parkinson's disease (PD), and amyotrophic lateral sclerosis (ALS), further reinforcing its role in neurodegeneration (Hedskog et al., 2013; Prause et al., 2013)[2]. Similarly[1], D2R activation has been associated with neuroprotective effects, including modifying intracellular signaling pathways that enhance neuronal survival. Therefore[1], the interaction between Sig1R and D2R may collaboratively enhance neuroprotection (Pinton et al., 2015)[2]. Herein[1,26], ART elevated the level[26] of both Sig1R and D2R, indicating a neuroprotective effect. Our findings are in agreement with previous reports on other anti-inflammatory and neuroprotective agents investigated in 3-NP models. For[1] instance, curcumin was shown to attenuate 3-NP–induced neurotoxicity by suppressing proinflammatory cytokines and enhancing antioxidant defenses (D'Egidio et al., 2023)[2]. Similarly[1], silymarin improved behavioral and biochemical outcomes by modulating oxidative stress pathways and protecting mitochondria (Haddadi et

al., 2024).[2] Melatonin[1] has also been reported to exert protective effects by reducing lipid peroxidation and inhibiting neuroinflammatory signaling (D'Egidio et al., 2023).[2] Consistent[1] with these studies, ART in the present work significantly reduced neuroinflammation, oxidative stress, and necroptotic signaling, thereby reinforcing its potential as a neuroprotective compound against 3-NP-induced striatal injury.

Collectively, 3-NP intoxication led to a noticeable increase in HMGB1/TLR4/p38-MAPK/NF-κB signaling, accompanied by a reduction in Sig1R and D2R levels, in agreement with previous reports. Cotreatment[1] with ART significantly attenuated activation[17] of the HMGB1/TLR4/p38-MAPK/NF-κB axis and restored Sig1R and D2R expression. These[1] findings extend existing evidence on the involvement of this inflammatory signaling pathway in Artemisinin's biological actions by contextualizing its modulation within a 3-NP-induced Huntington's disease-like model. Importantly[1], the observed effects should be interpreted as preclinical and pathway-associated neuroprotective responses in an acute experimental setting, rather than definitive evidence of therapeutic efficacy or CNS-targeted disease modification. Further[1] mechanistic and translational studies will be required to determine the relevance of these pathway associations in chronic or genetic models of Huntington's disease.

While the present study provides novel insights into the neuroprotective potential of ART against 3-NP-induced Huntington's disease-like pathology, certain limitations should be acknowledged. First[1], the treatment period was relatively short, and the employed 3-NP paradigm represents an acute/subacute toxin-based model that may not adequately reflect the long-term therapeutic efficacy of ART or the progressive, genetic nature of Huntington's disease. Second[1], although our findings demonstrate modulation

of HMGB1/TLR4/NF-κB signaling and attenuation of necroptosis, causal mechanistic verification using genetic knockdown approaches or selective pharmacological inhibitors (e.g., TLR4 antagonists, HMGB1-neutralizing antibodies, or necroptosis inhibitors) was not performed due to the scope of the approved experimental protocol. In[1] addition, pharmacokinetic profiling and direct assessment of BBB penetration were not conducted; therefore, CNS exposure and target engagement of ART under the present dosing regimen were not experimentally confirmed. Moreover[1], key pathogenic mechanisms implicated in Huntington's disease, including dysregulation of autophagy, classical apoptotic pathways (e.g., caspase-3 activation and Bax/Bcl-2 balance), and synaptic integrity markers such as PSD-95, were not examined. These[1] constraints limit definitive mechanistic and translational interpretation, and the findings should not be overinterpreted as evidence of established CNS-targeted therapeutic efficacy. Future[1] studies employing prolonged treatment regimens, pathway-specific mechanistic interventions, pharmacokinetic and BBB analyses, and validation in chronic or genetic Huntington's disease models will be essential to confirm the therapeutic relevance, optimal dosing, bioavailability, and long-term safety of ART before clinical translation can be considered.

1.	*. Artemisinin; . This; . 3-NP; . Biochemically; . Treatment; . At; . Collectively; . These; . Clinically; . One; . To; . The; . Furthermore; . However; . Preclinical; . Apart; . Moreover; . Therefore; . Code; . Efforts; . Located; . They; . A; . Group; . In; . Treatments; . Following; . After; . Fo...*	Text inconsistencies	Correctness
2.	*(Cho, 2012); (Kumar et al., 2010); (Gao et al., 2015); (Wu et al., 2010; Ibrahim & Abdel Rasheed, 2022); (Mustafa et al., 2024); (Wei et al., 2023); (Zhao et al., 2019; Lin et al., 2021); (Zhao et al., 2022); (Lawlor et al., 2015; Ali et al., 2021); (Zhao et al., 2019); (Mustafa et al., 2021); (Gua...*	Citation style options	Correctness
3.	~~to them~~	Paragraph can be improved	Clarity
4.	~~normal~~ → standard	Word choice	Engagement
5.	*Following the evaluations, the animals were put down, their striatum was removed, rinsed with ice-cold saline, flash-frozen in liquid nitrogen, and then stored at -80°C for future analysis.*	Paragraph can be improved	Clarity
6.	~~. Scheme~~ → —scheme	Incomplete sentences	Delivery
7.	*The tests were carried out over two days with a 2-hour break in between each day during the light cycle.*	Paragraph can be improved	Clarity
8.	*We used the camera to monitor two behavioral variables: the number of squares traversed by each rat (ambulation frequency) and the rate at which each rat stood up on its hind legs (rearing frequency).*	Paragraph can be improved	Clarity
9.	~~days in a row~~ → consecutive days	Paragraph can be improved	Clarity
10.	~~Bio BASIC Inc.~~ → Bio-Rad	Paragraph can be improved	Clarity

11.	*Protein concentrations of 10 μg for each sample were deposited onto PVDF membranes after SDS-PAGE separation and were blocked with 5% BSA.*	Paragraph can be improved	Clarity
12.	~~negative~~ → opposing	Word choice	Engagement
13.	~~normal~~ → standard	Word choice	Engagement
14.	*The immunoreactivity of striatal GFAP was evaluated through immunostaining to assess the extent of astrocyte activation.*	Paragraph can be improved	Clarity
15.	*Statistical analysis using one-way ANOVA revealed a significant difference between the groups (GFAP: F (4, 45) = 97.07, P < 0.0001).*	Paragraph can be improved	Clarity
16.	*This reduction was even more pronounced with a higher dose of ART (100 mg), which significantly decreased GFAP expression by 56.05% (P < 0.0001) relative to the 3-NP group.*	Paragraph can be improved	Clarity
17.	*activation; Activation*	Text inconsistencies	Correctness
18.	~~cognitive~~ → mental	Word choice	Engagement
19.	*Notably, elevated levels of oxidative stress markers, such as malondialdehyde (MDA) and 8-hydroxydeoxyguanosine (8-OHdG), are consistently observed in neurodegenerative brain regions of patients with Huntington's disease (Klepac et al., 2007).*	Paragraph can be improved	Clarity
20.	*In line with a previous study, ART treatment significantly ameliorated oxidative stress, as indicated by increased Nrf2 levels and downregulation of 8-OHdG (Deng et al., 2025).*	Paragraph can be improved	Clarity
21.	~~highlighting its ability to attenuate lipid peroxidation and~~	Paragraph can be improved	Clarity
22.	*This*	Intricate text	Clarity

23.	*An inflammatory reaction is triggered by massive amounts of DAMPs released from necroptotic cells (Zhang et al., 2017).*	Paragraph can be improved	Clarity
24.	*Activated RIPK1 subsequently activates the downstream target RIPK3, which polymerizes and converges with RIPK1 to form a necrosome complex.*	Paragraph can be improved	Clarity
25.	3-NP-treated	Paragraph can be improved	Clarity
26.	*Herein, ART elevated the level of both Sig1R and D2R, indicating a neuroprotective effect.*	Paragraph can be improved	Clarity
